# Global, regional, and national trends in Guillain–Barré syndrome burden from 1990 to 2021 and projections to 2041

**DOI:** 10.1097/MD.0000000000049163

**Published:** 2026-06-12

**Authors:** Fanyu Zhang, Zhengqiao Bao, Jingxian Wang, Shengguang Zhang, Ping Gao, Peiyang Zhou

**Affiliations:** a Department of Neurology, Xiangyang No. 1 People’s Hospital, Hubei University of Medicine, Xiangyang, Hubei, China; b Department of Neurology, Postgraduate Union Training Base of Xiangyang No. 1 People’s Hospital, School of Medicine, Wuhan University of Science and Technology, Xiangyang, Hubei, China; c Department of Radiology, Xiangyang No. 1 People’s Hospital, Hubei University of Medicine, Xiangyang, Hubei, China.

**Keywords:** COVID-19 pandemic, Global Burden of Disease, Guillain–Barré syndrome, sociodemographic disparities

## Abstract

This ecological study was based on secondary, population-level data from the Global Burden of Disease 2021 study. We systematically assessed the burden of Guillain–Barré syndrome (GBS) from 1990 to 2021 at global, regional, and national levels, and projected trends through 2041. Temporal trends were evaluated using joinpoint regression to calculate the average annual percentage change. Future trends up to 2041 were projected using autoregressive integrated moving average models. In 2021, the global number of prevalent GBS cases reached 471,850, with an age-standardized prevalence rate of 5.91 per 100,000, a 229% increase since 1990 (average annual percentage change: 3.34). Substantial regional disparities were observed, and frontier analysis indicated an inverse correlation between the sociodemographic index (SDI) and age-standardized rates. The disease burden was higher in males than in females, with peak prevalence occurring among children aged 5 to 9 years. The coronavirus disease 2019 pandemic was temporally associated with a marked increase in GBS burden in 2021, coinciding with a shift in the burden toward low-SDI regions. Projections suggest that the age-standardized prevalence rate will continue to rise through 2041, disproportionately affecting resource-limited areas. The global burden of GBS increased markedly from 1990 to 2021, with a notable rise after 2019 that was temporally associated with the coronavirus disease 2019 pandemic. Addressing this growing burden will warrant targeted interventions, strengthened surveillance, and equitable allocation of resources, particularly in low-SDI populations.

## 1. Introduction

Guillain–Barré syndrome (GBS) is an acute immune-mediated polyneuropathy, often triggered by infections.^[1]^ A variety of etiologies have been identified,^[2,3]^ including *Campylobacter jejuni,* influenza virus, diarrheal diseases, and upper respiratory tract infections.^[4]^ Recently, studies have suggested potential associations between GBS and novel factors such as Zika virus, severe acute respiratory syndrome coronavirus (SARS-CoV-2) infection, and Coronavirus Disease 2019 (COVID-19) vaccination.^[5–7]^ These emerging links reflect a shifting etiological landscape and highlight the need for an updated epidemiological assessment of GBS triggers.

As the leading cause of acute flaccid paralysis worldwide, GBS can progress to severe clinical outcomes, including quadriplegia and respiratory failure.^[8]^ Despite advances in intensive care, about 20% of patients require mechanical ventilation, and mortality can reach up to 5% in certain settings.^[9,10]^ These severe outcomes impose a substantial socioeconomic burden, indicating the importance of understanding the epidemiological trends of GBS. Unlike most autoimmune diseases, GBS exhibits a clear male predominance, with a male-to-female ratio of 1.5:1.^[8,9,11]^ Although high-income regions report an annual incidence of 1 to 2 cases per 100,000 population,^[9]^ data from low- and middle-income countries (LMICs) remain limited and heterogeneous.^[12]^ Furthermore, comprehensive analyses of GBS trends across geographic, demographic, and socioeconomic contexts are lacking, obscuring the actual burden of the disease and hindering the development of targeted interventions for at-risk populations.

While recent studies have offered valuable insights into the burden of GBS, many have not fully addressed emerging etiological drivers, particularly the impact of the COVID-19 pandemic.^[11,13]^ The pandemic has not only coincided with the emergence of COVID-19 as a potential associated etiological category for GBS in the Global Burden of Disease (GBD) framework but has also disrupted healthcare systems, potentially affecting case detection and management worldwide. Moreover, previous studies have generally lacked predictive modeling to inform targeted prevention and management strategies.^[13]^ Therefore, a thorough assessment of GBS disease burden is urgently warranted to address these gaps.

To that end, we conducted a comprehensive analysis of the global, regional, and national burden of GBS from 1990 to 2021 using data from the GBD study 2021, stratified by cause, sex, age, and sociodemographic index (SDI). We also examined the relationship between GBS burden and SDI and performed frontier analysis to identify efficiency gaps between countries. Furthermore, we projected GBS trends through 2041 using an autoregressive integrated moving average (ARIMA) model.

## 2. Methods

### 2.1. Data collection and GBS burden evaluation

This study was based on population-level estimates from the GBD 2021 database. Data for this study were obtained from the publicly available GBD 2021 Results Tool and included the prevalence of GBS, years lived with disability (YLDs), and their corresponding age-standardized indicators, the age-standardized prevalence rate (ASPR) and the age-standardized YLD rate (ASYR), for the period 1990 to 2021. While prevalence reflects the overall population burden of GBS, YLDs quantify nonfatal health loss by combining case numbers with disability weights. Age-standardized measures (ASPR and ASYR) account for differences in population age structures, enabling meaningful comparisons across countries and over time. In the GBD framework, GBS is defined according to standardized case definitions based on clinical diagnosis and International Classification of Diseases coding, as described in previous GBD methodological studies.

To evaluate potential etiological factors contributing to GBS, 7 categories were included based on the GBD 2021 classification: COVID-19, lower respiratory infections, other unspecified infectious diseases, Zika virus infection, diarrheal diseases, other neurological disorders, and upper respiratory infections. Temporal trends were assessed by calculating the average annual percentage change (AAPC), derived from a log-linear regression model to quantify both the magnitude and direction of changes in disease burden over time. In addition, to examine the relationship between GBS burden and socioeconomic development, the SDI, a composite measure reflecting per capita income, educational attainment, and total fertility rate, was incorporated. All estimates in the present study, including prevalence, YLDs, ASPR, ASYR, and the trend indicator AAPC, were reported with 95% uncertainty intervals (UIs), calculated from the 2.5th and 97.5th percentiles of 1000 posterior draws to ensure statistical robustness. Because this study used only de-identified, publicly available data, Institutional Review Board approval was not required.

### 2.2. Frontier analysis

To assess whether the global burden of GBS is associated with sociodemographic development, we employed frontier analysis. This quantitative method identifies the lowest achievable age-standardized rates (ASRs) given a country’s development status, as measured by the SDI. Detailed explanations of SDI calculation methods in GBD research have been provided in previous studies.^[14,15]^ For analysis, countries and regions included in the GBD study were grouped into 5 SDI quintiles: low, low-middle, middle, high-middle, and high SDI. Frontier analysis was then applied as previously described, with the frontier representing the minimum achievable prevalence and YLDs for each country or region based on its SDI.

### 2.3. Statistical analysis and prediction scope

The AAPC was calculated using a log-linear regression model to quantify temporal trends. Joinpoint regression analysis, a widely used method in epidemiological research^[16]^ known for its parsimony and strong model fit, was applied to identify potential inflection points in the trends. Future trends from 2022 to 2041 were projected using ARIMA models. Model selection was based on the akaike information criterion. Model adequacy was assessed using residual diagnostics, including visual inspection of residual plots and tests for autocorrelation. (Table S2 and Table S3, Supplemental Digital Content) Forecasts were interpreted cautiously given the uncertainty surrounding future epidemiological changes.^[17]^

## 3. Results

### 3.1. Long-term trends of GBS burden

**Figure 1.**
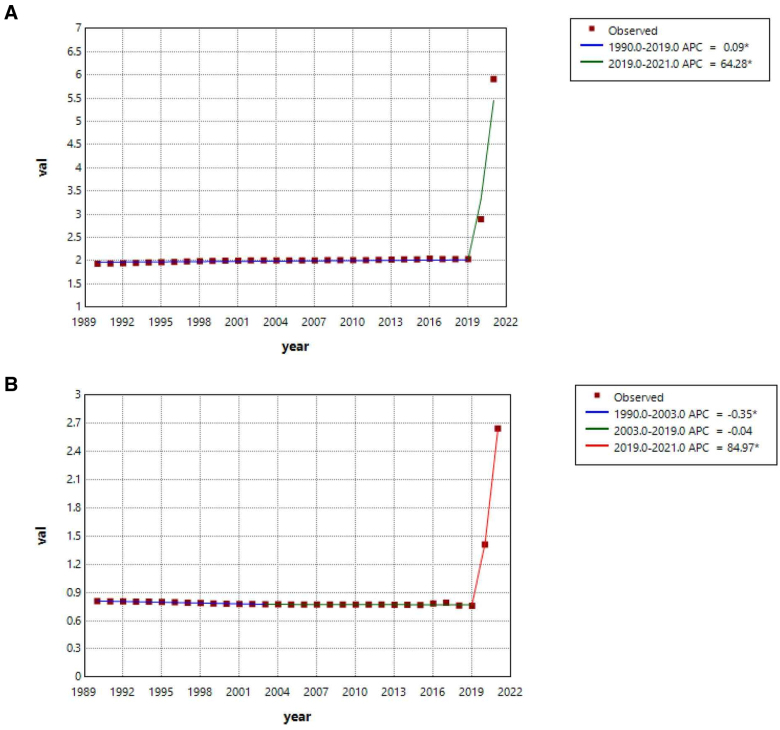
Joinpoint regression analysis of global trends in the burden of GBS, 1990 to 2021. (A) Trends in the ASPR of GBS. (B) Trends in the ASYR of GBS. Joinpoint regression was used to evaluate temporal trends. All rates are expressed per 100,000 population. APC = annual percentage change, ASPR = age-standardized prevalence rate, ASYR = age-standardized years lived with disability rate, GBS = Guillain–Barré syndrome.

From 1990 to 2021, the global burden of GBS increased steadily, with a marked acceleration after 2019 (Fig. 1). In 2021, the number of prevalent GBS cases reached 471,850 (95% UI: 389,186–554,144), corresponding to an ASPR of 5.91 per 100,000 (95% UI: 4.87–6.97) (Table 1). This represented a 229% increase since 1990, with an AAPC of 3.34 (95% confidence interval [CI]: 3.26–3.42). Similarly, YLDs due to GBS reached 139,639 (95% UI: 90,387–202,387), with an ASYR of 1.75 per 100,000 (95% UI: 1.12–2.54), also reflecting a 229% increase (AAPC 3.34, 95% CI: 3.26–3.42).

**Table 1:** YLDs and prevalence due to Guillain-Barre syndrome in 2021, the percentage change and the AAPC in the ASRs per 100,000, by location.

	Prevalence (95% UI)				YLD (95% UI)			
	N (95% UI)	ASRs per 100,000 (95% UI)	Percentage change in ASRs between 1990 and 2021	AAPC (95% UI)	N (95% UI)	ASRs per 100,000 (95% UI)	Percentage change in ASRs between 1990 and 2021	AAPC (95% UI)
Global	471,850 (389,187–554,145)	5.91 (4.87–6.97)	229 (209–241)	3.34 (3.26–3.42)	139,639 (90,387–202,387)	1.75 (1.12–2.54)	229 (216–238)	3.34 (3.26–3.42)
East Asia	9894 (7378–12,857)	0.63 (0.48–0.82)	22 (17–24)	0.26 (0.09–0.43)	2929 (1798–4469)	0.19 (0.12–0.29)	22 (19–24)	0.26 (0.09–0.43)
Southeast Asia	32,889 (27,154–39,008)	4.69 (3.89–5.55)	176 (151–196)	2.63 (2.57–2.70)	9735 (6143–14,041)	1.39 (0.88–2.0)	176 (160–181)	2.63 (2.57–2.70)
Oceania	431 (291–613)	3.21 (2.22–4.54)	175 (148–193)	0.28 (0.16–0.72)	127 (73–208)	0.95 (0.55–1.54)	175 (158–188)	0.28 (0.16–0.72)
Central Asia	6603 (5099–8254)	6.98 (5.39–8.72)	309 (304–308)	4.21 (4.09–4.13)	1953 (1205–2853)	2.06 (1.27–3.01)	308 (291–301)	4.21 (4.09–4.13)
Central Europe	9278 (7596–11,047)	7.24 (5.83–8.66)	432 (397–464)	4.61 (4.47–4.75)	2742 (1719–4001)	2.14 (1.33–3.12)	431 (411–425)	4.61 (4.47–4.75)
Eastern Europe	19,125 (15,420–23,116)	8.43 (6.75–10.21)	383 (368–398)	4.53 (4.53–4.54)	5643 (3601–8478)	2.49 (1.58–3.75)	382 (382–384)	4.53 (4.53–4.54)
High-income Asia Pacific	11,630 (9759–13,834)	6.34 (5.28–7.74)	12 (7–18)	0.38 (0.35–0.41)	3443 (2267–4991)	1.88 (1.22–2.77)	12 (8–16)	0.38 (0.35–0.41)
Australasia	591 (466–737)	1.62 (1.31–2.0)	52 (47–54)	0.94 (0.92–0.96)	175 (111–259)	0.48 (0.31–0.7)	52 (53–56)	0.94 (0.92–0.96)
Western Europe	19,783 (16,854–23,082)	3.81 (3.21–4.46)	159 (143–179)	2.91 (2.75–3.07)	5856 (3889–8260)	1.13 (0.74–1.61)	160 (146–172)	2.91 (2.75–3.07)
Southern Latin America	4314 (3604–5150)	6.05 (5.02–7.31)	91 (87–95)	1.71 (1.65–1.77)	1278 (809–1849)	1.79 (1.14–2.62)	91 (88–89)	1.71 (1.65–1.77)
High-income North America	31,481 (27,535–35,800)	6.93 (5.96–7.9)	126 (111–142)	2.08 (2.03–2.13)	9317 (6135–12,990)	2.05 (1.34–2.92)	125 (113–130)	2.08 (2.03–2.13)
Caribbean	1877 (1471–2335)	3.88 (3.03–4.82)	100 (95–99)	1.86 (1.38–2.34)	556 (351–798)	1.15 (0.73–1.65)	100 (91–98)	1.86 (1.38–2.34)
Andean Latin America	5868 (4614–7276)	8.94 (7.05–11.05)	298 (282–301)	3.87 (3.71–4.02)	1736 (1082–2571)	2.64 (1.65–3.9)	297 (288–304)	3.87 (3.71–4.02)
Central Latin America	23,966 (20,089–28,098)	9.49 (7.97–11.13)	142 (123–154)	2.43 (2.27–2.58)	7095 (4485–10,211)	2.81 (1.78–4.05)	142 (131–152)	2.43 (2.27–2.58)
Tropical Latin America	15,982 (13,016–19,371)	6.76 (5.48–8.21)	529 (499–559)	5.14 (4.82–5.47)	4724 (3008–6863)	2.0 (1.27–2.91)	528 (506–533)	5.14 (4.82–5.47)
North Africa and Middle East	38,857 (30,710–47,881)	6.47 (5.13–7.94)	361 (334–372)	4.55 (4.49–4.60)	11,497 (7168–16,761)	1.91 (1.19–2.79)	360 (332–361)	4.55 (4.49–4.60)
South Asia	155,150 (126,682–185,328)	8.48 (6.93–10.11)	287 (259–306)	3.77 (3.76–3.77)	45,945 (28,849–68,526)	2.51 (1.58–3.75)	287 (277–295)	3.77 (3.76–3.77)
Central Sub-Saharan Africa	11,132 (7868–13,862)	8.97 (6.38–11.12)	413 (371–377)	5.2 (5.18–5.21)	3291 (1974–4829)	2.65 (1.6–3.84)	411 (394–402)	5.2 (5.18–5.21)
Eastern Sub-Saharan Africa	32,607 (26,110–39,434)	8.33 (6.78–10.04)	448 (396–470)	5.09 (5.02–5.16)	9640 (6076–14,105)	2.46 (1.55–3.58)	446 (416–467)	5.09 (5.02–5.16)
Southern Sub-Saharan Africa	6183 (4925–7480)	7.82 (6.24–9.42)	335 (303–351)	4.07 (4.06–4.08)	1830 (1136–2676)	2.31 (1.45–3.39)	335 (309–338)	4.07 (4.06–4.08)
Western Sub-Saharan Africa	34,208 (26,981–41,335)	7.79 (6.18–9.38)	312 (278–320)	4.62 (4.60–4.64)	10,130 (6419–14,958)	2.31 (1.48–3.4)	312 (299–328)	4.62 (4.60–4.64)

AAPC = average annual percent change, ASR = age-standardized rates, N = number of individuals, UI = uncertainty interval, YDL = years lived with disability.

Growth trajectories varied substantially across regions and countries. Tropical Latin America experienced the most significant increase in ASPR between 1990 and 2021 (+529%, 95% UI: 499–559; AAPC: 5.14, 95% CI: 5.00–5.28), whereas the High-Income Asia Pacific region showed the smallest increase (+12%, 95% UI: 7–18; AAPC: 0.38, 95% CI: 0.35–0.41) (Table 1). At the national level, North Macedonia exhibited the most pronounced rise (+700%, 95% UI: 479–1022; AAPC: 6.32, 95% CI: 5.30–6.80), while New Zealand was the only country with a decline in ASPR (−7%, 95% UI: −13–0.00; AAPC: −0.22, 95% CI: −0.23–−0.21) (Table S1, Supplemental Digital Content). Sex disparities were also evident across most regions, with males generally showing higher ASPR and ASYR than females (Figs. S1A and S1B Supplemental Digital Content).

### 3.2. Regional and national disparities of GBS burden in 2021

**Figure 2.**
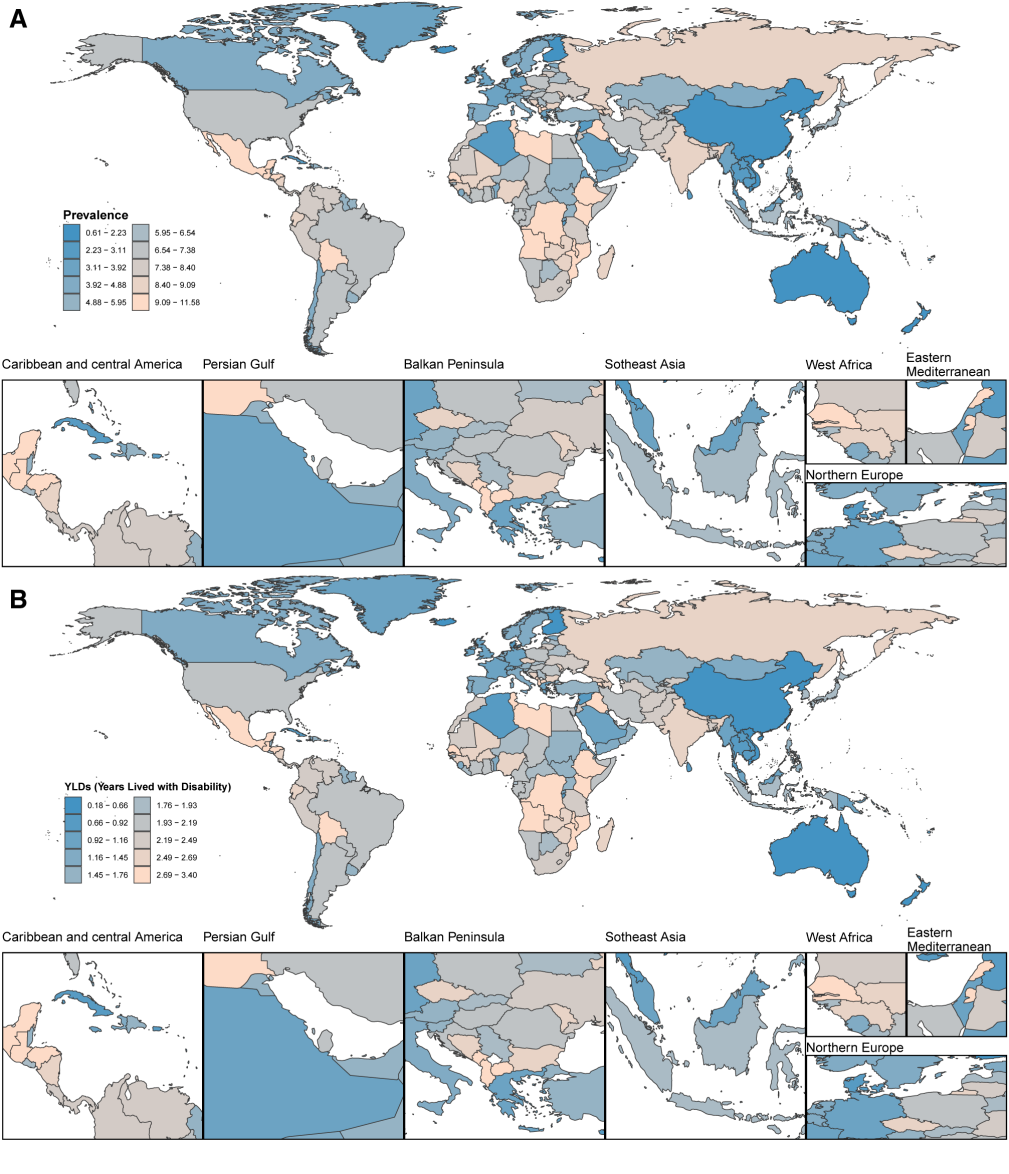
Global distribution of the burden of GBS by country in 2021. (A) ASPR of GBS. (B) ASYR of GBS. All rates are expressed per 100,000 population. ASPR = age-standardized prevalence rate, ASYR = age-standardized years lived with disability rate, GBS = Guillain–Barré syndrome, YLD = years lived with disability.

In 2021, substantial geographical heterogeneity in the burden of GBS was evident (Fig. 2, Table 1, and Table S1, Supplemental Digital Content). At the regional level, the highest ASPR values were observed in Central Latin America (9.49 per 100,000; 95% UI: 7.97–11.13), Central Sub-Saharan Africa (8.97 per 100,000; 95% UI: 6.38–11.12), and Andean Latin America (8.94 per 100,000; 95% UI: 7.05–11.05) (Table 1, Figs. S1C and S1D, Supplemental Digital Content). The lowest ASPR values were observed in East Asia (0.63 per 100,000; 95% UI: 0.48–0.82) and Australasia (1.62 per 100,000; 95% UI: 1.31–2.00). At the national level, the highest burden was reported in North Macedonia (11.47 per 100,000; 95% UI: 8.21–14.48), Bolivia (11.34 per 100,000; 95% UI: 5.39–14.17), and Iraq (11.24 per 100,000; 95% UI: 7.59–14.84), whereas China (0.62 per 100,000; 95% UI: 0.47–0.80) and the Democratic People’s Republic of Korea (0.84 per 100,000; 95% UI: 0.65–1.08) had the lowest burden (Table S1, Supplemental Digital Content Fig. 2A).

Of note, regional disparities in YLDs closely mirrored the heterogeneity observed in ASPR (Fig. 2B, Table S1, Supplemental Digital Content). At the regional level, the highest ASYR values were observed in Central Latin America (2.81 per 100,000; 95% UI: 1.78–4.05), Central Sub-Saharan Africa (2.65 per 100,000; 95% UI: 1.60–3.84), and Andean Latin America (2.64 per 100,000; 95% UI: 1.65–3.90). The lowest ASYR values were reported in East Asia (0.19 per 100,000; 95% UI: 0.12–0.29) and Australasia (0.48 per 100,000; 95% UI: 0.31–0.70). At the national level, the highest burden was observed in North Macedonia (11.47 per 100,000; 95% UI: 8.21–14.48), Bolivia (11.34 per 100,000; 95% UI: 8.39–14.17), and Iraq (11.24 per 100,000; 95% UI: 7.59–14.64), while China (0.62 per 100,000; 95% UI: 0.47–0.80) and the Democratic People’s Republic of Korea (0.84 per 100,000; 95% UI: 0.65–1.08) had the lowest burden (Table S1, Supplemental Digital Content).

### 3.3. Age- and sex-specific patterns of GBS

**Figure 3.**
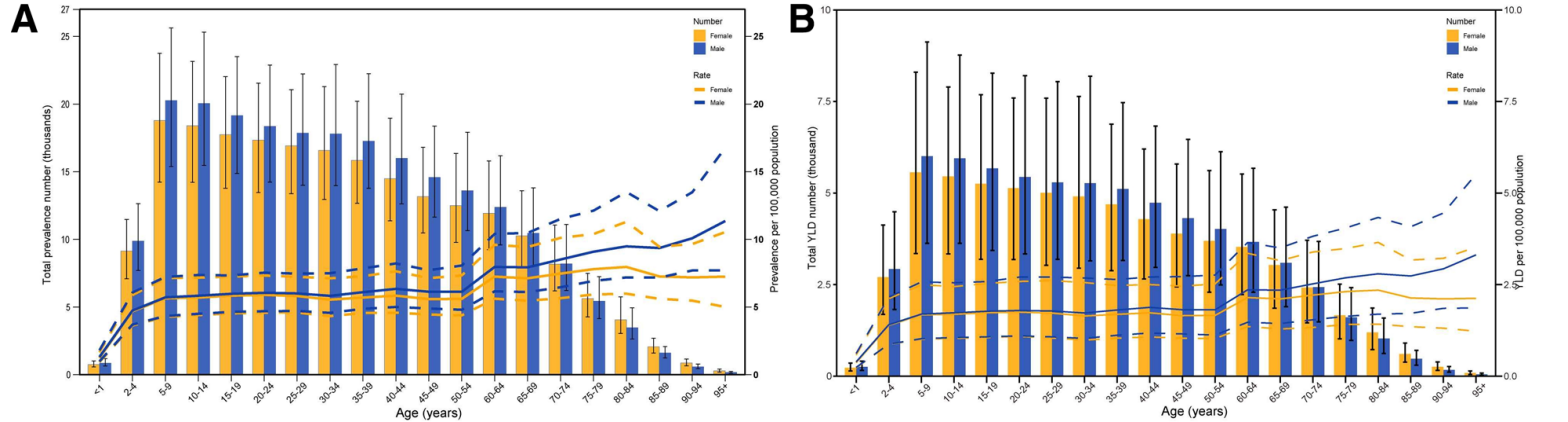
Global age-specific burden of GBS by sex in 2021. (A) Number of prevalent cases and prevalence rates by age and sex. (B) Number of YLD cases and YLD rates by age and sex. Bars indicate absolute numbers, and lines indicate rates per 100,000 population. The left y-axis represents the absolute number, and the right y-axis represents the rate. GBS = Guillain–Barré syndrome, YLD = years lived with disability.

Globally, the ASPR of GBS in 2021 showed distinct patterns by age and sex. Prevalence peaked in the 5- to 9-year age group and declined through middle age (Fig. 3A). Males consistently had higher prevalence than females across most age strata, although this sex disparity gradually narrowed with advancing age, reaching parity in the 70- to 74-year group before reversing in older populations. The burden of disease measured by YLDs largely mirrored these prevalence patterns across age and sex (Fig. 3B). Notably, ASYR values in the oldest age groups were proportionally higher than prevalence rates, likely reflecting greater disability severity per case among the elderly.

### 3.4. COVID-19 as the leading associated etiological category in 2021

**Figure 4.**
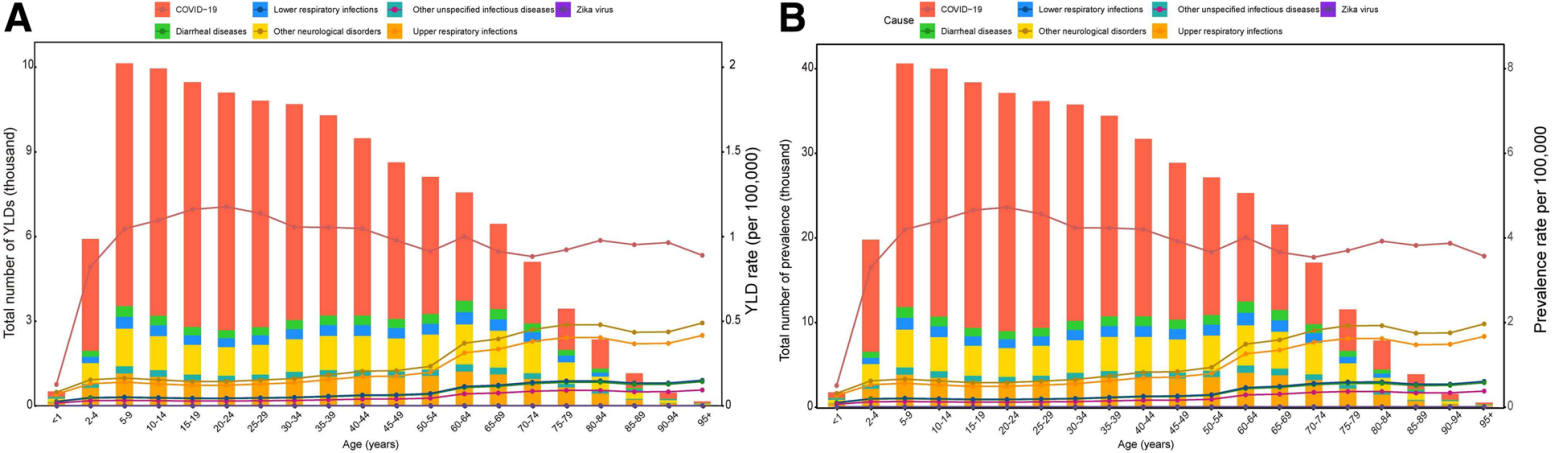
Burden of GBS attributable to underlying causes by age globally in 2021. (A) Number of YLD cases and YLD rates attributable to each underlying cause by age. (B) Number of prevalent cases and prevalence rates attributable to each underlying cause by age. Bars indicate absolute numbers, and lines indicate rates per 100,000 population. The left y-axis represents the absolute number, and the right y-axis represents the rate. COVID-19 = coronavirus disease 2019, GBS = Guillain–Barré syndrome, YLD = years lived with disability.

In 2021, COVID-19 emerged as the leading associated category within the GBD 2021 attribution framework of GBS-related YLDs (Fig. 4A). Age-specific analyses revealed distinct profiles: while all-cause GBS YLDs peaked in the 5- to 9-year age group, COVID-19-related YLDs uniquely peaked in the 20- to 24-year age group. After 2020, COVID-19 was temporally associated with a marked increase in GBS burden, surpassing all other infectious categories by 2021 within the GBD attribution framework (Fig. 4A, Fig. S2B, Supplemental Digital Content). Importantly, patterns in prevalence closely mirrored those observed in YLDs (Fig. 4B, Fig. S2A, Supplemental Digital Content).

### 3.5. Association of GBS burden with SDI

The burden of GBS was strongly associated with SDI, with pronounced regional and national variations. Globally, ASPR and ASYR remained relatively stable until 2019, followed by a sharp increase across all SDI quintiles. Notably, high-SDI regions experienced the most rapid post-2019 increases (Figs. S3A, Supplemental Digital Content and S3B, Supplemental Digital Content).

**Figure 5.**
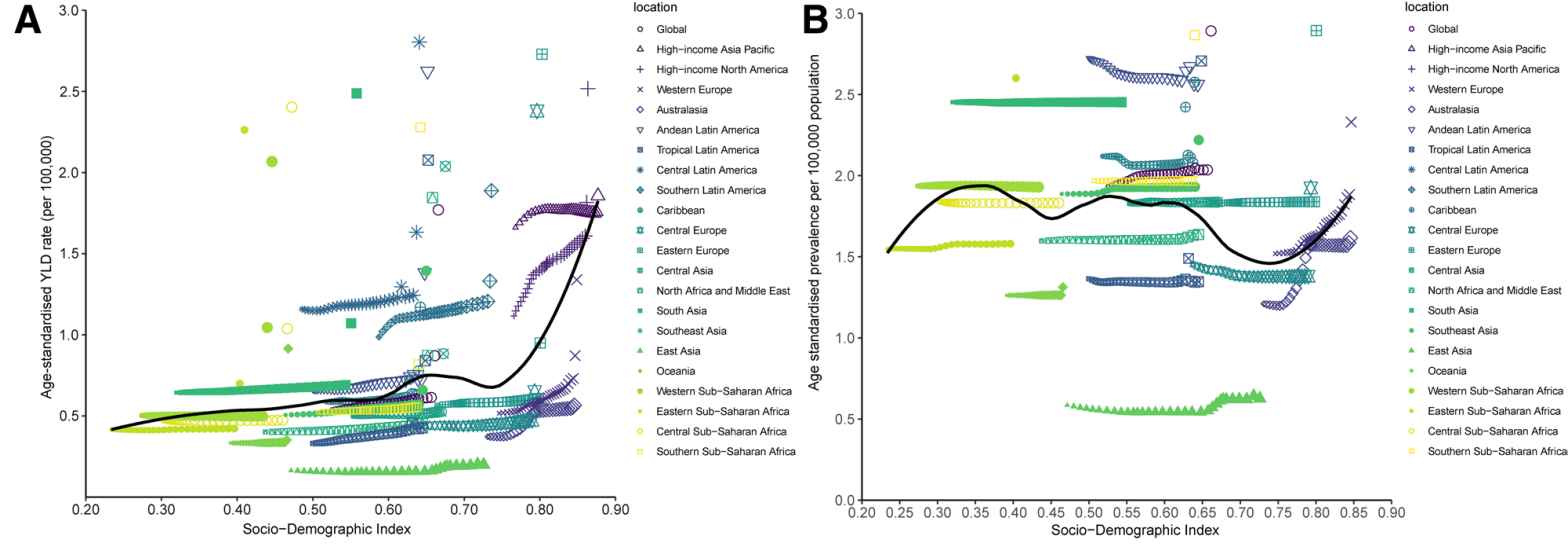
Trends in the burden of GBS globally and across 21 GBD regions by sociodemographic development, 1990 to 2021. (A) ASYR in 21 GBD regions. (B) ASPR globally and in 21 GBD regions. All rates are expressed per 100,000 population. ASPR = age-standardized prevalence rate, ASYR = age-standardized years lived with disability rate, GBD = Global Burden of Disease, GBS = Guillain–Barré syndrome, SDI = sociodemographic index, YLD = years lived with disability.

Regionally, SDI was positively correlated with ASYR. Eastern Sub-Saharan Africa, Eastern Europe, South Asia, and Andean Latin America exhibited higher-than-expected burdens relative to their SDI levels. In contrast, East Asia, Australasia, Oceania, Western Europe, and the Caribbean consistently showed lower-than-expected burdens (Fig. 5).

At the national level, a nonlinear relationship was observed: ASYR decreased with increasing SDI up to 0.5, then fluctuated thereafter. Countries with disproportionately high burdens included North Macedonia, Albania, Montenegro, and Bolivia, whereas China, the Democratic People’s Republic of Korea, and Taiwan reported lower-than-expected values (Fig. S4, Supplemental Digital Content).

### 3.6. Frontier analysis

**Figure 6.**
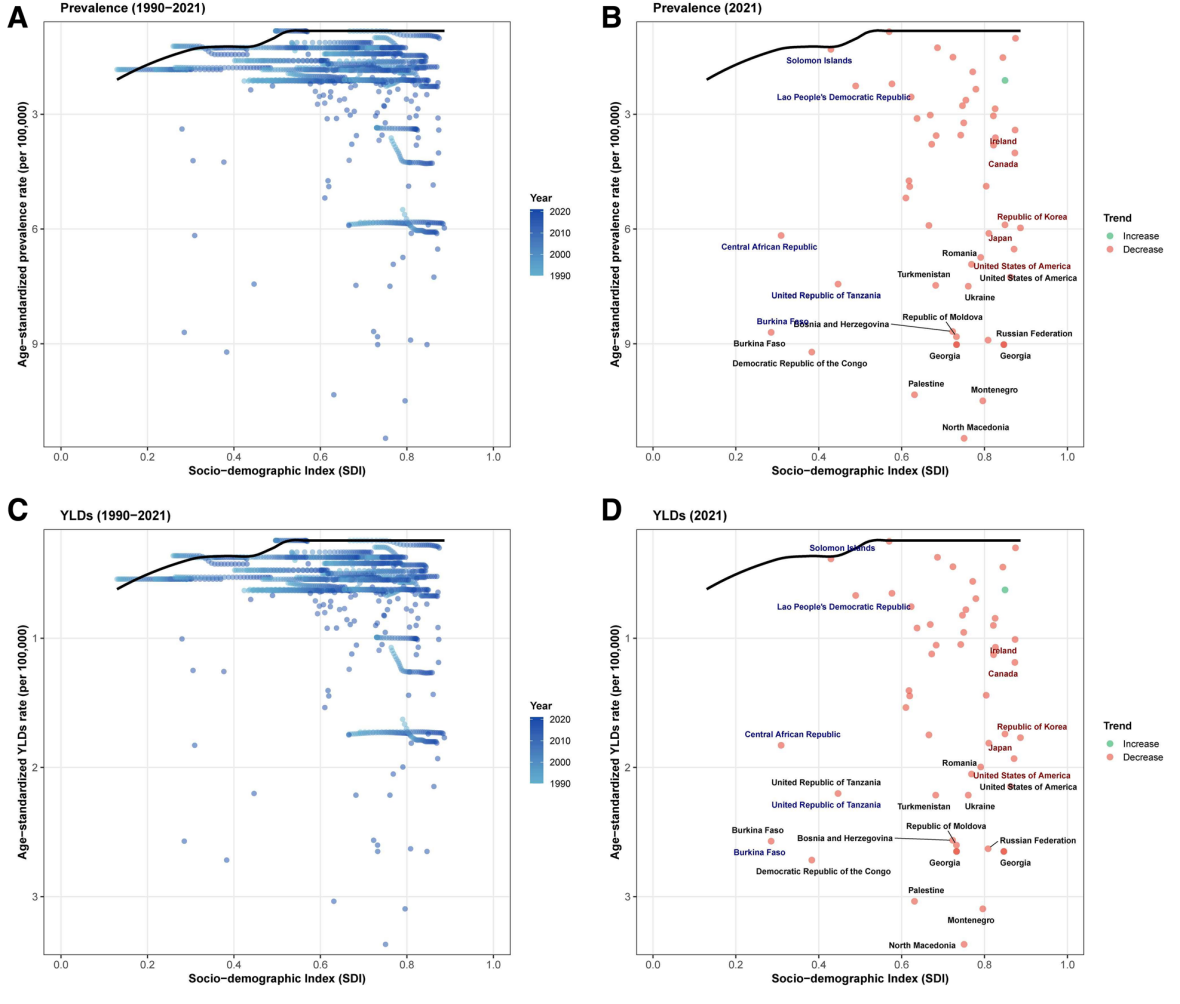
Frontier analysis of the burden of GBS in relation to sociodemographic development. The black solid line represents the frontier, indicating the lowest achievable burden at a given level of the SDI. Each point represents a country or territory. The vertical distance from the frontier (deviation) reflects the gap between the observed burden and the optimal level, indicating the potential for improvement. (A, B) show the relationship between SDI and ASPR, and (C, D) show the relationship between SDI and ASYR. The color gradient represents temporal changes from 1990 (light) to 2021 (dark). All rates are expressed per 100,000 population. ASPR = age-standardized prevalence rate, ASYR = age-standardized years lived with disability rate, GBS = Guillain–Barré syndrome, SDI = sociodemographic index, YLD = years lived with disability.

Next, frontier analysis further demonstrated the relationship between SDI and GBS ASRs (ASPR and ASYR) (Figs. 6A, 6C). Both ASPR and ASYR were inversely associated with SDI, decreasing progressively as SDI increased from 0 to 1, suggesting that greater development is associated with a lower GBS burden. Using GBD 2021 data and SDI, we calculated the effective differences between each country or region and the frontier, identifying some countries that achieved the best possible health outcomes relative to their SDI levels. Among these, the Solomon Islands and the Lao People’s Democratic Republic consistently had ASPR and ASYR values for GBS close to the frontier curve. In contrast, 15 countries, including North Macedonia, Montenegro, and Palestine, exhibited substantial deviations from the frontier in both ASPR and ASYR (Figs. 6B, 6D).

### 3.7. Projected trends to 2041

**Figure 7.**
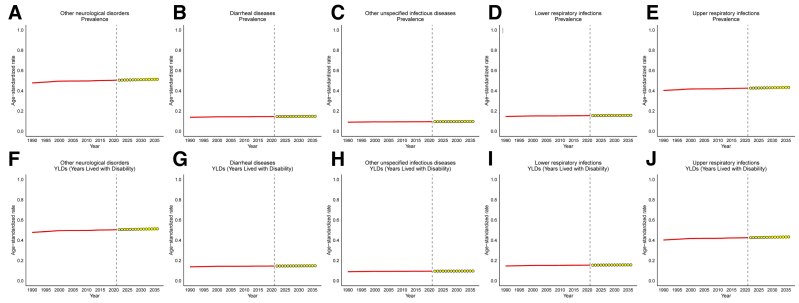
Forecasted trends in the global burden of GBS by underlying causes, 2022 to 2041. Forecasts were generated using ARIMA models based on historical data from 1990 to 2021. (A–E) Projected YLD rates for different underlying causes. (F–J) Projected prevalence rates for different underlying causes. Lines represent predicted values, and shaded areas represent 95% prediction intervals. All rates are expressed per 100,000 population. ARIMA = autoregressive integrated moving average, GBS = Guillain–Barré syndrome, YLDs = years lived with disability.

Projections indicate that ASPR and ASYR of GBS will continue to rise slightly across all etiologies over the next 2 decades (Figs. 7A–7J). Although the impact of COVID-19 is expected to wane, upper respiratory infections and other neurological disorders are projected to remain the primary contributors to GBS burden by 2041.

## 4. Discussion

The present study examined the global burden of GBS across geographic and temporal scales and identified a significant upward trend in ASRs (ASPR and ASYR) from 1990 to 2021. Specifically, ASRs increased sharply between 2019 and 2021, temporally associated with the COVID-19 pandemic, which disrupted long-term gains in life expectancy. Male predominance was observed across most age groups, with a distinct peak in childhood (5–9 years) and a reversal of the sex ratio in older populations. Projections indicate that the burden of GBS will continue to rise through 2041, with upper respiratory infections and other neurological disorders expected to remain the leading associated categories, rather than COVID-19. Our findings are broadly consistent with previous global analyses showing substantial regional heterogeneity in GBS burden while extending the literature by incorporating GBD 2021 data and forward projections to 2041.

The sustained increase in the global burden of GBS from 1990 to 2021 may reflect multiple factors, including greater disease awareness, improved diagnostic capabilities, and enhanced surveillance systems over time.^[4]^ In addition, evolving environmental exposures and changes in circulating infectious agents, such as *C. jejuni,* Zika virus, and respiratory pathogens, may have contributed to the long-term rise in incidence.^[18–20]^ Of note, the sharp increase in GBS burden across most countries and territories after 2019 may be partly linked to the global spread of COVID-19, which emerged as the leading associated etiological category of GBS in 2021 within the GBD attribution framework.^[21,22]^ Studies have also suggested that pandemics may elevate GBS risk through both SARS-CoV-2 infection and mass vaccination.^[23–25]^ For instance, the Lombardy region of Italy reported a 2.6-fold increase in GBS incidence during March-April 2020 compared with the same period in 2019, consistent with the findings of this study.^[26]^ Although SARS-CoV-2 vaccines are generally well tolerated, certain types, particularly adenovirus vector-based vaccines, have been reported in some studies to be associated with a small increased risk of GBS.^[27]^ Several mechanisms have been proposed in previous studies, including molecular mimicry and immune-mediated cross-reactivity,which can provoke localized inflammatory or autoimmune reactions in the peripheral nervous system.^[28]^ These findings highlight the need for resilient and adaptable public health systems to minimize the neurological impact of emerging infectious diseases.

Notably, the parallel trends of YLDs and ASPR suggest that regions with high GBS prevalence also bear a greater disability burden. This may reflect overlapping factors, including higher exposure to triggering infections (e.g., *C. jejuni,* norovirus, or SARS-CoV-2), variations in healthcare infrastructure affecting timely diagnosis and treatment, and differences in acute management and rehabilitation capacity.^[29–31]^ Moreover, the COVID-19 pandemic after 2019 may have been associated with wider disparities, particularly in regions with preexisting weaknesses in healthcare systems.^[26]^ Studies have shown that both COVID-19 infection and vaccination are associated with an increased risk of GBS, and the pandemic’s impact on healthcare access and rehabilitation services may have been associated with longer disability duration and higher YLD estimates, particularly in low- and middle-income regions.^[29,32,33]^ This consistent spatial pattern reflects the need for integrated surveillance of both prevalence and long-term disability to guide resource allocation and targeted interventions in high-burden areas.

We observed pronounced geographical heterogeneity in GBS burden in 2021. ASPR was notably higher in regions including Latin America (Central and Andean) and Central Sub-Saharan Africa, while Australasia and East Asia exhibited the lowest rates. This regional variation aligns with the IGOS-1000 study,^[34]^ which found that although the distribution of preceding infections was similar across continents, the clinical phenotype associated with *C. jejuni* infection varied significantly by region. The high burden in Latin America and Sub-Saharan Africa may be closely linked to the elevated prevalence of specific pathogens in these regions. For example, gastrointestinal infections such as *C. jejuni* are well-established triggers of GBS, and their incidence is typically higher in areas with limited sanitation infrastructure.^[35]^ Furthermore, outbreaks of arboviruses such as the Zika virus in Latin America have been linked to surges in GBS cases.^[36,37]^ Limited healthcare resources in these regions may also contribute to underdiagnosis and delayed treatment, exacerbating the long-term disability burden. By contrast, the burden of GBS in East Asia, particularly China, is among the lowest globally. This low burden may reflect multiple factors, including differences in genetic background^[38]^ and population susceptibility to specific GBS-triggering infections.^[39]^ Second, during the COVID-19 pandemic, widespread non-pharmaceutical interventions in East Asia,^[40,41]^ such as mask-wearing and social distancing, substantially reduced the transmission of multiple infectious diseases, including respiratory and gastrointestinal viruses, which may have been associated with a short-term decrease in GBS incidence.^[42]^ Thus, China’s low burden reflects the combined influence of long-term regional epidemiological characteristics and the specific public health measures implemented during this period.

Our analysis also highlights the demographic characteristics of GBS. Although it affects all age groups, the disease consistently shows male predominance and a notable burden among older adults. The male predominance observed in our study is consistent with the sex-specific incidence patterns reported by Sejvar et al,^[43]^ who found a significantly higher risk for males (relative risk 1.78, 95% CI 1.36–2.33). The relatively greater disability burden observed among older adults may be attributable to immunosenescence, a higher prevalence of comorbidities, and greater exposure to triggering infections.^[42]^ This finding underscores the need for enhanced preventive strategies and improved access to healthcare for these populations.Notably, GBS prevalence peaks among children aged 5 to 9 years, which may be associated with heightened susceptibility to specific pathogens such as *C. jejuni* or Norovirus.^[44,45]^ This observation aligns with recent reports of Norovirus and *C. jejuni* detection in pediatric GBS outbreaks in India and other regions.^[45]^ Male predominance in individuals under 70 to 75 years may reflect sex-based differences in immune responses,^[46,47]^ whereas the reversal in the oldest age groups could result from longer female lifespan, reduced hormonal protection, and gene-environment interactions.^[48]^ Further studies are needed to clarify these mechanisms.

Another key finding of our study is the complex, nonlinear relationship between socioeconomic development and GBS burden. Countries with a low SDI, such as North Macedonia and Bolivia, bear a disproportionately high burden of GBS. This disparity may result from a combination of factors, including inadequate targeted prevention programs, limited healthcare resources, and shortages of specialized medical personnel.^[12,49]^This is further illustrated by the IGOS-1000 study,^[34]^ which demonstrated that patients in Bangladesh had significantly lower rates of immunomodulatory treatment and worse functional outcomes compared to patients in high-income regions. In contrast, high-SDI regions benefit from greater resource availability, such as widespread access to neurophysiological testing and cerebrospinal fluid analysis, which facilitate earlier and more accurate diagnosis.^[50]^ The stark inequity in neurological care is further highlighted by the fact that the density of neurology professionals per 100,000 people is roughly 70 times higher in high-income countries than in LMICs.^[11]^ These findings underscore the urgent need to strengthen health systems and establish specialized neurology care programs in resource-limited settings.

To inform future healthcare planning, we used ARIMA models to project the etiology-specific burden of GBS through 2041. Given data limitations beyond 2021 and uncertainty regarding the long-term trajectory of COVID-19,^[11]^ we conservatively assumed that COVID-19’s contribution to GBS burden would decline linearly to 0. Even so, projections indicate a persistent, modest increase in overall ASPR and ASYR driven by other etiologies over the next 2 decades. This shows the need for sustained disease surveillance, renewed efforts to prevent and control known infectious triggers, and proactive strategic planning to allocate healthcare resources for managing the anticipated rise in GBS burden.

Several limitations of this study should be acknowledged. First, GBD estimates rely heavily on statistical modeling in regions with sparse or low-quality data, which may introduce uncertainty and potential bias, particularly in LMICs with limited surveillance systems. In addition, diagnostic coding for GBS may not be entirely accurate, and limited data collection in low-SDI settings may further contribute to the underestimation of cases. During the COVID-19 pandemic, the reallocation of healthcare resources toward infectious disease management may also have resulted in underreporting or delays in reporting GBS cases. Second, the use of uniform disability weights across all etiologies may not fully capture differences in long-term disability severity, particularly for COVID-19-related GBS, which may follow distinct clinical trajectories.^[20]^ Third, our ARIMA forecasting models were based on historical trends and did not account for potential future changes, such as the introduction of novel therapies, shifts in vaccination policies, or the emergence of new pathogens, which may affect the accuracy of long-term projections. Finally, because this was an ecological study based on secondary population-level data, individual-level risk factors, such as metabolic comorbidities and other clinical characteristics, could not be incorporated, which may limit the comprehensiveness of the etiological assessment.

## 5. Conclusions

This study provides a comprehensive assessment of the global, regional, and national burden of GBS from 1990 to 2021, revealing a marked and sustained increase in both prevalence and YLDs. Following 2019, the COVID-19 pandemic was temporally associated with a marked increase in GBS burden, with a disproportionate shift toward low-SDI regions, highlighting the vulnerability of health systems to emerging pathogens. Projections through 2041 indicate a continued rise in GBS burden, with upper respiratory infections and other neurological disorders remaining the leading associated categories, even as the direct impact of COVID-19 diminishes.These findings emphasize the urgent need for targeted public health interventions, strengthened global surveillance, and equitable allocation of healthcare resources, particularly in high-burden, resource-limited regions.

## Acknowledgments

We are thankful to all the academics that have participated in and contributed to the GBD study.

## Author contributions

**Conceptualization:** Jingxian Wang.

**Data curation:** Peiyang Zhou.

**Formal analysis:** Fanyu Zhang, Ping Gao.

**Funding acquisition:** Peiyang Zhou.

**Methodology:** Fanyu Zhang.

**Project administration:** Zhengqiao Bao, Ping Gao, Peiyang Zhou.

**Resources:** Shengguang Zhang.

**Visualization:** Fanyu Zhang, Zhengqiao Bao.

**Writing – original draft:** Fanyu Zhang, Zhengqiao Bao, Jingxian Wang, Shengguang Zhang, Ping Gao.

**Writing – review & editing:** Fanyu Zhang, Zhengqiao Bao.







**Figure:**
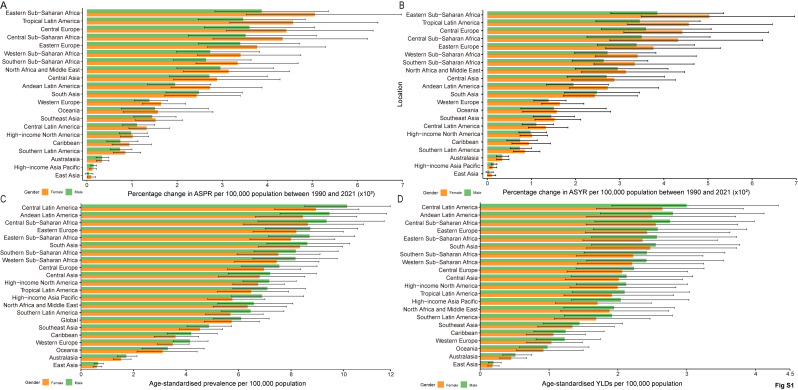


**Figure:**
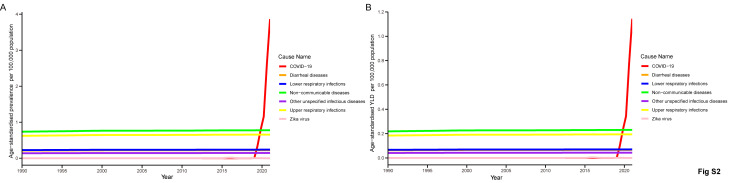


**Figure:**
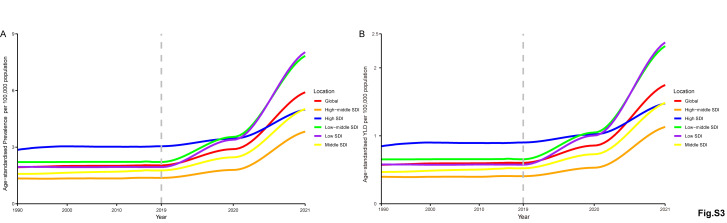


**Figure:**
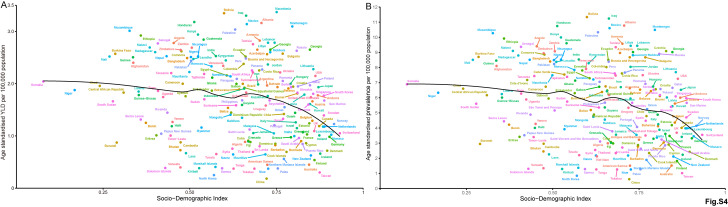


## References

[1] Leonhard SE Mandarakas MR de Assis Aquino Gondim F. Evidence based guidelines. Diagnosis and management of Guillain-Barre syndrome in ten steps. Medicina (B Aires). 2021;81:817–36. 34633957

[2] Abara WE Gee J Marquez P. Reports of guillain-barré syndrome after COVID-19 vaccination in the United States. JAMA Netw Open. 2023;6:e2253845. 36723942 10.1001/jamanetworkopen.2022.53845 PMC9892957

[3] Bellanti R Rinaldi S. Guillain‐barré syndrome: a comprehensive review. Eur J Neurol. 2024;31:e16365. 38813755 10.1111/ene.16365 PMC11235944

[4] Bragazzi NL Kolahi AA Nejadghaderi SA. Global, regional, and national burden of Guillain–Barré syndrome and its underlying causes from 1990 to 2019. J Neuroinflammation. 2021;18:264. 34763713 10.1186/s12974-021-02319-4 PMC8581128

[5] Cagnazzo F Arquizan C Derraz I. Neurological manifestations of patients infected with the SARS-CoV-2: a systematic review of the literature. J Neurol. 2021;268:2656–65. 33125542 10.1007/s00415-020-10285-9 PMC7597753

[6] Censi S Bisaccia G Gallina S Tomassini V Uncini A. Guillain–Barré syndrome and COVID-19 vaccination: a systematic review and meta-analysis. J Neurol. 2024;271:1063–71. 38233678 10.1007/s00415-024-12186-7 PMC10896967

[7] Doets AY Verboon C Van Den Berg B. Regional variation of guillain-barré syndrome. Brain. 2018;141:2866–77. 30247567 10.1093/brain/awy232

[8] Ferrari AJ Santomauro DF Aali A. Global incidence, prevalence, years lived with disability (YLDs), disability-adjusted life-years (DALYs), and healthy life expectancy (HALE) for 371 diseases and injuries in 204 countries and territories and 811 subnational locations, 1990–2021: a systematic analysis for the global burden of disease study 2021. Lancet. 2024;403:2133–61. 38642570 10.1016/S0140-6736(24)00757-8 PMC11122111

[9] Filosto M Cotti Piccinelli S Gazzina S. Guillain-Barré syndrome and COVID-19: an observational multicentre study from two italian hotspot regions. J Neurol Neurosurg Psychiatry. 2021;92:751–6. 33158914 10.1136/jnnp-2020-324837

[10] Alqahtani S Al-Dorzi HM Arishi H. Characteristics and outcomes of patients with Guillain-Barré syndrome who were admitted to the intensive care unit: a retrospective observational study. J Int Med Res. 2024;52:3000605241306655. 39719074 10.1177/03000605241306655 PMC11683809

[11] Finsterer J. Exacerbating Guillain-Barré syndrome eight days after vector-based COVID-19 vaccination. Case Rep Infect Dis. 2021;2021:3619131. 34055430 10.1155/2021/3619131 PMC8123983

[12] Florian IA Lupan I Sur L Samasca G Timiș TL. To be, or not to be… guillain-barré syndrome. Autoimmun Rev. 2021;20:102983. 34718164 10.1016/j.autrev.2021.102983

[13] Fragiel M Miró O Llorens P. Incidence, clinical, risk factors and outcomes of Guillain‐Barré in covid‐19. Ann Neurol. 2021;89:598–603. 33295021 10.1002/ana.25987

[14] Yang T Lei Y Liao L. Global, regional, and national burden of liver cancer due to non-alcoholic steatohepatitis and non-alcoholic fatty liver disease, 1990-2021: a multi-model trend analysis and forecasting study. Hepatol Int. 2025;19:619–32. 39939576 10.1007/s12072-025-10782-x

[15] Bai Z Han J An J. The global, regional, and national patterns of change in the burden of congenital birth defects, 1990-2021: an analysis of the global burden of disease study 2021 and forecast to 2040. EClinicalMedicine. 2024;77:102873. 39416384 10.1016/j.eclinm.2024.102873 PMC11474384

[16] Lai P Cai W Liu F Hong C Tan W Zhao Z. Trend analysis of changes in the incidence of tuberculosis in Shenzhen from 2010 to 2020 based on the Joinpoint regression model. BMC Public Health. 2025;25:4081. 41272518 10.1186/s12889-025-25118-5 PMC12639698

[17] Frontera JA Tamborska AA Doheim MF. Neurological events reported after COVID‐19 vaccines: an analysis of vaccine adverse event reporting system. Ann Neurol. 2022;91:756–71. 35233819 10.1002/ana.26339 PMC9082459

[18] Kaakoush NO Castaño-Rodríguez N Mitchell HM Man SM. Global epidemiology of campylobacter infection. Clin Microbiol Rev. 2015;28:687–720. 26062576 10.1128/CMR.00006-15 PMC4462680

[19] Le Vu S Bertrand M Botton J. Risk of guillain-barré syndrome following COVID-19 vaccines: a nationwide self-controlled case series study. Neurology. 2023;101:e2094–102. 37788935 10.1212/WNL.0000000000207847 PMC10663040

[20] Leonhard SE Mandarakas MR Gondim FAA. Diagnosis and management of guillain–barré syndrome in ten steps. Nat Rev Neurol. 2019;15:671–83. 31541214 10.1038/s41582-019-0250-9 PMC6821638

[21] Leonhard SE Papri N Querol L Rinaldi S Shahrizaila N Jacobs BC. Guillain–barré syndrome. Nat Rev Dis Primer. 2024;10:97. 10.1038/s41572-024-00580-4 39702645

[22] Levison LS Thomsen RW Markvardsen LK Christensen DH Sindrup SH Andersen H. Pediatric guillain-barré syndrome in a 30-year nationwide cohort. Pediatr Neurol. 2020;107:57–63. 32192820 10.1016/j.pediatrneurol.2020.01.017

[23] Minamino M Miyamoto K Kuwahara M. Characteristics of Guillain-Barre syndrome in super-elderly individuals. J Neurol. 2023;270:2191–6. 36645487 10.1007/s00415-023-11567-8

[24] Morita D Mukhopadhyay AK Chowdhury G. Genomic epidemiology and genetic characteristics of clinical Campylobacter species cocirculating in West Bengal, India, 2019, using whole genome analysis. Antimicrob Agents Chemother. 2025;69:e0110824. 39629976 10.1128/aac.01108-24 PMC11784092

[25] Ngiam JN Cheong CWS Leow AST. Stress hyperglycaemia is associated with poor functional outcomes in patients with acute ischaemic stroke after intravenous thrombolysis. QJM. 2022;115:7–11. 32810234 10.1093/qjmed/hcaa253

[26] Ottaviani D Boso F Tranquillini E. Early guillain-barré syndrome in coronavirus disease 2019 (COVID-19): a case report from an italian COVID-hospital. Neurol Sci. 2020;41:1351–4. 32399950 10.1007/s10072-020-04449-8 PMC7216127

[27] García-Grimshaw M Galnares-Olalde JA Bello-Chavolla OY. Incidence of Guillain-Barre syndrome following SARS-CoV-2 immunization: Analysis of a nationwide registry of recipients of 81 million doses of seven vaccines. Eur J Neurol. 2022;29:3368–79. 35841212 10.1111/ene.15504 PMC9349509

[28] Shahrizaila N Lehmann HC Kuwabara S. Guillain-barré syndrome. Lancet. 2021;397:1214–28. 33647239 10.1016/S0140-6736(21)00517-1

[29] Gào X Zhao C Yang J. Impact of COVID-19 vaccination coverage on global disability burden of Guillain-Barré syndrome. NPJ Vaccines. 2025;10:182. 40753095 10.1038/s41541-025-01239-1 PMC12318086

[30] Papri N Doets AY Luijten L. Prediction of respiratory failure and prolonged mechanical ventilation in Guillain-Barré syndrome: a prospective cohort study in Bangladesh. J Peripher Nerv Syst. 2024;29:428–40. 39581760 10.1111/jns.12673

[31] Kalita J Mahajan R Bhoi SK Misra UK. Outcome of Guillain-Barre syndrome with bulbar palsy. Am J Med Sci. 2024;368:621–7. 38992752 10.1016/j.amjms.2024.07.010

[32] Park J Bae J. Guillain-Barré syndrome during the COVID-19 era: a nationwide study of hospitalized cases in South Korea. Medicine (Baltimore). 2025;104:e41677. 39993100 10.1097/MD.0000000000041677 PMC11856989

[33] Chen Y Lai K Lee Y Liao Y. Incidence and characteristics of Guillain-Barré syndrome in Taiwan before and during the COVID-19 pandemic: a 12-year single-center experience. J Chin Med Assoc. 2025;88:503–12. 40442888 10.1097/JCMA.0000000000001251 PMC12637126

[34] Leonhard SE Van Der Eijk AA Andersen H. An international perspective on preceding infections in Guillain-Barré syndrome: the IGOS-1000 cohort. Neurology. 2022;99:e1299–313. 35981895 10.1212/WNL.0000000000200885

[35] Heimesaat M Backert S Alter T Bereswill S. Human campylobacteriosis-a serious infectious threat in a one health perspective. In: Backert S, ed. Fighting Campylobacter Infections: Towards a One Health Approach. Vol. 431. Springer Nature; 2021:1–23. 10.1007/978-3-030-65481-8_1 33620646

[36] Tewedaj ZD Huluka DK Kebede YT. A retrospective analysis of the clinical profile and factors associated with mortality and poor hospital outcomes in adult Guillain-Barre syndrome patients. Sci Rep. 2024;14:15520. 38969647 10.1038/s41598-024-65265-0 PMC11226644

[37] Acero-Garces D Zuluaga-Lotero D Ortiz-Muñoz D. Long-term outcomes of patients affected by Guillain-Barré syndrome in Colombia after the Zika virus epidemic. J Neurol Sci. 2024;463:123140. 39047509 10.1016/j.jns.2024.123140 PMC11338696

[38] Bae JS Yuki N Kuwabara S. Guillain-Barré syndrome in Asia. J Neurol Neurosurg Psychiatry. 2014;85:907–13. 24357682 10.1136/jnnp-2013-306212

[39] Thomma RCM Halstead SK de Koning LC. Large-scale profiling of antibody reactivity to glycolipids in patients with Guillain-Barré syndrome. Brain. 2025;148:4000–15. 40096525 10.1093/brain/awaf102 PMC12588681

[40] Lee HJ Hwang BS Im SH Mun SK Chang M. Clinical effects of non-pharmaceutical interventions for COVID-19 on other nationally notifiable infectious diseases in South Korea. Korean J Intern Med. 2024;39:823–32. 39135523 10.3904/kjim.2023.501 PMC11384254

[41] Geng MJ Zhang HY Yu LJ. Changes in notifiable infectious disease incidence in China during the COVID-19 pandemic. Nat Commun. 2021;12:6923. 34836947 10.1038/s41467-021-27292-7 PMC8626444

[42] Choi SA Hwang J Lim BC Chae SA. Incidence of Guillain-Barre syndrome in South Korea during the early COVID-19 pandemic. Front Neurol. 2023;14:1125455. 36895908 10.3389/fneur.2023.1125455 PMC9989167

[43] Sejvar JJ Baughman AL Wise M Morgan OW. Population incidence of Guillain-Barré syndrome: a systematic review and meta-analysis. Neuroepidemiology. 2011;36:123–33. 21422765 10.1159/000324710 PMC5703046

[44] Priyadarshini D Anuhya V Mahapatra A. Clinico-epidemiological profile and prediction of outcome in children with Guillain-Barre syndrome. Ital J Pediatr. 2025;51:179–90. 40483522 10.1186/s13052-025-02037-0 PMC12145608

[45] Lavania M Sharma V Meena VK. Norovirus genomes detected from the Guillain-Barré syndrome (GBS) cases in a community outbreak in Pune, India, 2025. J Infect. 2025;91:106604. 40897243 10.1016/j.jinf.2025.106604

[46] McCombe PA Hardy TA Nona RJ Greer JM. Sex differences in Guillain Barré syndrome, chronic inflammatory demyelinating polyradiculoneuropathy and experimental autoimmune neuritis. Front Immunol. 2022;13:1038411. 36569912 10.3389/fimmu.2022.1038411 PMC9780466

[47] Wallace RL Cribb DM Bulach DM. Campylobacter jejuni ST50, a pathogen of global importance: a comparative genomic analysis of isolates from Australia, Europe and North America. Zoonoses Public Health. 2021;68:638–49. 34041858 10.1111/zph.12853

[48] Willison AG Pawlitzki M Lunn MP Willison HJ Hartung HP Meuth SG. SARS-CoV-2 vaccination and neuroimmunological disease: a review. JAMA Neurol. 2024;81:179–86. 38227318 10.1001/jamaneurol.2023.5208

[49] Winkler AS. The growing burden of neurological disorders in low-income and middle-income countries: priorities for policy making. Lancet Neurol. 2020;19:200–2. 31813849 10.1016/S1474-4422(19)30476-4

[50] Yang J Li J Lai S. Uncovering two phases of early intercontinental COVID-19 transmission dynamics. J Travel Med. 2020;27:taaa200. 33094347 10.1093/jtm/taaa200 PMC7665593

