# Supplementary material for: Global, regional, and national trends in Guillain–Barré syndrome burden from 1990 to 2021 and projections to 2041
Source: Medicine (Baltimore). 2026 Jun 12;105(24):e49163. doi: 10.1097/MD.0000000000049163 (PMC13268433; doi:10.1097/MD.0000000000049163)
Supplement: Supplementary file 3 [file medi-105-e49163-s003.docx]

| Table S1. The prevalence and YLDs of GBDS in 2021 for both sexes and all countries, with AAPC (%) from 1990 to 2021 (generated from data available from: ghdx.healthdata.org/gbd-results-tools/) | | | | | | | | | |  |  |  |
| --- | --- | --- | --- | --- | --- | --- | --- | --- | --- | --- | --- | --- |
|  | YLD (95% UI) |  |  |  |  |  | Prevalence (95% UI) |  |  |  |  |  |
|  | 1990 |  | 2021 |  |  |  | 1990 |  | 2021 |  |  |  |
| location | Counts (95% UI) | ASR per 100,000 (95% UI) | Counts (95% UI) | ASR per 100,000 (95% UI) | % Change in counts from 1990 to 2021 | AAPC in ASR from 1990 to 2021 | Counts (95% UI) | ASR per 100,000 (95% UI) | Counts (95% UI) | ASR per 100,000 (95% UI) | % Change in counts from 1990 to 2021 | AAPC in ASR from 1990 to 2021 |
| Afghanistan | 41 (26 - 63) | 0.47 (0.30 - 0.73) | 651 (342 - 1027) | 2.40 (1.25 - 3.79) | 395 (235- 599) | 5.45 (5.37 - 5.56) | 141 (108 - 181) | 1.60 (1.24 - 2.02) | 2197 (1375 - 2824) | 8.10 (5.23 - 10.34) | 394 (235- 600) | 5.45 (5.37 - 5.56) |
| Albania | 12 (8 - 19) | 0.42 (0.27 - 0.63) | 91 (53 - 141) | 3.16 (1.83 - 4.78) | 775 (470- 1180) | 6.13 (5.08 - 6.59) | 43 (33 - 56) | 1.42 (1.11 - 1.80) | 312 (218 - 394) | 10.78 (7.46 - 13.54) | 788 (507- 1149) | 6.13 (5.08 - 6.59) |
| Algeria | 100 (62 - 155) | 0.47 (0.30 - 0.73) | 381 (231 - 581) | 0.89 (0.54 - 1.35) | 117 (68- 196) | 1.96 (1.83 - 2.02) | 338 (258 - 449) | 1.60 (1.24 - 2.02) | 1287 (937 - 1722) | 2.99 (2.20 - 3.97) | 117 (68- 196) | 1.96 (1.83 - 2.02) |
| American Samoa | 0 (0 - 0) | 0.37 (0.23 - 0.58) | 0 (0 - 0) | 0.45 (0.28 - 0.68) | 34 (16- 59) | 0.49 (0.31 - 0.60) | 0 (0 - 0) | 1.26 (0.98 - 1.59) | 0 (0 - 0) | 1.51 (1.17 - 1.88) | 34 (16- 59) | 0.49 (0.31 - 0.60) |
| Andorra | 0 (0 - 0) | 0.53 (0.34 - 0.81) | 1 (1 - 2) | 1.78 (1.11 - 2.65) | 258 (173- 381) | 3.91 (3.78 - 3.98) | 1 (0 - 1) | 1.78 (1.39 - 2.23) | 5 (4 - 7) | 6.01 (4.55 - 7.70) | 259 (173- 381) | 3.91 (3.78 - 3.98) |
| Angola | 48 (29 - 73) | 0.55 (0.35 - 0.83) | 811 (412 - 1236) | 2.78 (1.43 - 4.20) | 430 (219- 691) | 4.75 (3.79 - 5.22) | 162 (124 - 218) | 1.84 (1.43 - 2.31) | 2742 (1710 - 3598) | 9.40 (5.86 - 12.29) | 430 (219- 691) | 4.75 (3.79 - 5.22) |
| Antigua and Barbuda | 0 (0 - 0) | 0.62 (0.40 - 0.93) | 0 (0 - 1) | 0.96 (0.59 - 1.43) | 67 (40- 109) | 1.17 (0.67 - 1.59) | 1 (0 - 1) | 2.10 (1.67 - 2.62) | 3 (2 - 3) | 3.23 (2.51 - 3.99) | 67 (40- 109) | 1.17 (0.67 - 1.59) |
| Argentina | 365 (232 - 542) | 1.10 (0.70 - 1.63) | 930 (586 - 1359) | 1.96 (1.24 - 2.89) | 85 (57- 122) | 1.70 (1.30 - 2.04) | 1233 (990 - 1533) | 3.71 (2.96 - 4.59) | 3141 (2551 - 3898) | 6.63 (5.35 - 8.30) | 85 (57- 122) | 1.70 (1.30 - 2.04) |
| Armenia | 17 (11 - 26) | 0.54 (0.35 - 0.81) | 90 (50 - 137) | 2.82 (1.57 - 4.24) | 483 (272- 736) | 5.24 (4.88 - 5.41) | 60 (46 - 75) | 1.83 (1.45 - 2.30) | 307 (204 - 396) | 9.58 (6.35 - 12.34) | 484 (281- 728) | 5.24 (4.88 - 5.41) |
| Australia | 51 (32 - 74) | 0.30 (0.19 - 0.43) | 137 (85 - 205) | 0.45 (0.29 - 0.67) | 74 (49- 106) | 1.36 (1.33 - 1.38) | 173 (138 - 217) | 1.00 (0.81 - 1.25) | 462 (358 - 584) | 1.52 (1.20 - 1.91) | 74 (49- 106) | 1.36 (1.33 - 1.38) |
| Austria | 64 (41 - 91) | 0.68 (0.44 - 0.96) | 196 (125 - 279) | 1.66 (1.04 - 2.39) | 163 (132- 204) | 2.68 (2.22 - 3.02) | 217 (183 - 259) | 2.31 (1.97 - 2.74) | 663 (566 - 786) | 5.62 (4.72 - 6.75) | 163 (132- 204) | 2.68 (2.22 - 3.02) |
| Azerbaijan | 36 (23 - 55) | 0.54 (0.35 - 0.81) | 285 (151 - 456) | 2.65 (1.41 - 4.22) | 443 (217- 732) | 4.46 (3.30 - 5.02) | 123 (95 - 156) | 1.83 (1.45 - 2.30) | 966 (569 - 1325) | 8.96 (5.28 - 12.28) | 444 (214- 736) | 4.46 (3.30 - 5.02) |
| Bahamas | 1 (0 - 2) | 0.62 (0.40 - 0.93) | 4 (2 - 7) | 1.22 (0.72 - 1.87) | 122 (56- 202) | 1.91 (1.44 - 2.13) | 4 (3 - 6) | 2.10 (1.67 - 2.62) | 16 (11 - 21) | 4.12 (2.98 - 5.50) | 122 (56- 202) | 1.91 (1.44 - 2.13) |
| Bahrain | 2 (1 - 3) | 0.48 (0.30 - 0.73) | 29 (16 - 44) | 1.93 (1.13 - 2.93) | 379 (216- 628) | 4.25 (3.63 - 4.55) | 6 (5 - 9) | 1.61 (1.25 - 2.04) | 98 (67 - 135) | 6.54 (4.55 - 8.94) | 380 (216- 628) | 4.25 (3.63 - 4.55) |
| Bangladesh | 612 (379 - 921) | 0.66 (0.42 - 0.98) | 4077 (2307 - 6279) | 2.49 (1.41 - 3.82) | 341 (199- 540) | 4.15 (3.74 - 4.32) | 2067 (1613 - 2697) | 2.23 (1.79 - 2.79) | 13758 (9722 - 18423) | 8.40 (5.95 - 11.23) | 341 (199- 540) | 4.15 (3.74 - 4.32) |
| Barbados | 1 (0 - 2) | 0.62 (0.40 - 0.93) | 2 (1 - 4) | 0.82 (0.53 - 1.21) | 49 (30- 71) | 0.76 (0.42 - 1.05) | 5 (4 - 6) | 2.10 (1.67 - 2.62) | 9 (7 - 11) | 2.78 (2.25 - 3.35) | 49 (30- 71) | 0.76 (0.42 - 1.05) |
| Belarus | 54 (34 - 82) | 0.50 (0.31 - 0.76) | 195 (95 - 348) | 1.91 (0.93 - 3.42) | 302 (118- 568) | 3.94 (3.34 - 4.27) | 184 (142 - 232) | 1.68 (1.31 - 2.14) | 660 (353 - 1057) | 6.44 (3.49 - 10.34) | 302 (118- 568) | 3.94 (3.34 - 4.27) |
| Belgium | 60 (37 - 92) | 0.52 (0.33 - 0.79) | 171 (110 - 248) | 1.29 (0.83 - 1.87) | 146 (103- 215) | 2.87 (2.67 - 2.96) | 205 (157 - 260) | 1.76 (1.37 - 2.21) | 580 (465 - 705) | 4.36 (3.48 - 5.33) | 146 (103- 215) | 2.87 (2.67 - 2.96) |
| Belize | 1 (0 - 1) | 0.63 (0.41 - 0.94) | 6 (3 - 10) | 1.54 (0.78 - 2.45) | 164 (65- 292) | 2.36 (1.45 - 2.84) | 3 (2 - 4) | 2.13 (1.70 - 2.65) | 21 (13 - 31) | 5.19 (3.21 - 7.47) | 164 (65- 292) | 2.36 (1.45 - 2.84) |
| Benin | 22 (13 - 34) | 0.54 (0.34 - 0.83) | 149 (84 - 250) | 1.25 (0.71 - 2.10) | 137 (63- 256) | 2.44 (2.03 - 2.64) | 76 (58 - 103) | 1.83 (1.42 - 2.31) | 504 (337 - 735) | 4.22 (2.86 - 6.12) | 137 (63- 256) | 2.44 (2.03 - 2.64) |
| Bermuda | 0 (0 - 0) | 0.62 (0.40 - 0.93) | 0 (0 - 1) | 0.90 (0.58 - 1.31) | 71 (51- 99) | 1.03 (0.68 - 1.17) | 1 (0 - 1) | 2.11 (1.67 - 2.62) | 2 (1 - 2) | 3.04 (2.53 - 3.66) | 71 (51- 99) | 1.03 (0.68 - 1.17) |
| Bhutan | 3 (2 - 5) | 0.67 (0.42 - 0.99) | 5 (3 - 7) | 0.73 (0.47 - 1.07) | 25 (12- 42) | 0.25 (0.16 - 0.28) | 11 (9 - 15) | 2.25 (1.81 - 2.81) | 17 (14 - 22) | 2.45 (2.02 - 3.02) | 25 (12- 42) | 0.25 (0.16 - 0.28) |
| Bolivia | 40 (25 - 60) | 0.79 (0.52 - 1.18) | 386 (238 - 573) | 3.35 (2.08 - 4.94) | 411 (263- 604) | 4.73 (4.70 - 4.76) | 138 (110 - 174) | 2.68 (2.13 - 3.27) | 1309 (961 - 1644) | 11.34 (8.39 - 14.17) | 412 (269- 606) | 4.73 (4.70 - 4.76) |
| Bosnia and Herzegovina | 17 (11 - 27) | 0.40 (0.25 - 0.61) | 93 (53 - 142) | 2.56 (1.47 - 3.91) | 625 (387- 949) | 5.99 (4.88 - 6.51) | 59 (45 - 76) | 1.34 (1.05 - 1.71) | 317 (225 - 414) | 8.68 (6.08 - 11.34) | 627 (400- 943) | 5.99 (4.88 - 6.51) |
| Botswana | 6 (3 - 9) | 0.54 (0.34 - 0.82) | 44 (22 - 73) | 1.89 (0.97 - 3.13) | 294 (122- 549) | 0.48 (-0.26 - 1.24) | 20 (16 - 28) | 1.82 (1.42 - 2.29) | 149 (86 - 216) | 6.38 (3.73 - 9.18) | 294 (122- 549) | 0.48 (-0.26 - 1.24) |
| Brazil | 492 (311 - 741) | 0.40 (0.26 - 0.60) | 4575 (2903 - 6624) | 2.00 (1.27 - 2.90) | 527 (372- 739) | 5.19 (4.92 - 5.44) | 1662 (1291 - 2118) | 1.37 (1.07 - 1.72) | 15483 (12620 - 18827) | 6.75 (5.47 - 8.21) | 528 (374- 741) | 5.19 (4.92 - 5.44) |
| Brunei | 4 (2 - 6) | 1.74 (1.10 - 2.62) | 8 (5 - 12) | 1.81 (1.14 - 2.73) | 7 (-1- 17) | 0.12 (0.09 - 0.14) | 15 (11 - 19) | 5.88 (4.75 - 7.34) | 28 (22 - 35) | 6.11 (5.00 - 7.56) | 7 (-1- 17) | 0.12 (0.09 - 0.14) |
| Bulgaria | 40 (25 - 59) | 0.42 (0.27 - 0.63) | 196 (99 - 311) | 2.57 (1.32 - 4.12) | 528 (280- 819) | 4.97 (3.34 - 5.73) | 135 (105 - 171) | 1.42 (1.11 - 1.80) | 662 (404 - 878) | 8.67 (5.30 - 11.68) | 527 (263- 828) | 4.97 (3.34 - 5.73) |
| Burkina Faso | 45 (27 - 68) | 0.54 (0.34 - 0.83) | 518 (293 - 787) | 2.57 (1.46 - 3.87) | 381 (227- 599) | 4.96 (4.52 - 5.12) | 152 (117 - 203) | 1.83 (1.42 - 2.30) | 1751 (1208 - 2258) | 8.70 (6.01 - 11.24) | 382 (229- 599) | 4.96 (4.52 - 5.12) |
| Burundi | 22 (13 - 35) | 0.45 (0.28 - 0.68) | 103 (65 - 156) | 0.87 (0.55 - 1.32) | 96 (57- 157) | 1.85 (1.18 - 2.13) | 75 (57 - 98) | 1.51 (1.18 - 1.89) | 350 (264 - 447) | 2.95 (2.26 - 3.71) | 96 (57- 157) | 1.85 (1.18 - 2.13) |
| Cabo Verde | 1 (1 - 2) | 0.54 (0.34 - 0.82) | 12 (6 - 20) | 2.24 (1.18 - 3.61) | 356 (179- 569) | 4.39 (3.79 - 5.10) | 5 (4 - 7) | 1.82 (1.42 - 2.29) | 42 (25 - 56) | 7.54 (4.64 - 10.11) | 356 (180- 571) | 4.39 (3.79 - 5.10) |
| Cambodia | 46 (28 - 71) | 0.53 (0.33 - 0.79) | 145 (87 - 217) | 0.88 (0.53 - 1.31) | 89 (41- 148) | 1.31 (0.55 - 1.60) | 156 (120 - 205) | 1.78 (1.41 - 2.23) | 492 (355 - 630) | 2.96 (2.17 - 3.77) | 89 (41- 148) | 1.31 (0.55 - 1.60) |
| Cameroon | 49 (29 - 74) | 0.54 (0.35 - 0.83) | 574 (171 - 1002) | 2.00 (0.63 - 3.46) | 283 (24- 533) | 4.09 (3.82 - 4.22) | 166 (127 - 221) | 1.84 (1.43 - 2.31) | 1934 (642 - 2939) | 6.73 (2.27 - 10.18) | 282 (24- 533) | 4.09 (3.82 - 4.22) |
| Canada | 169 (110 - 247) | 0.57 (0.37 - 0.83) | 584 (368 - 870) | 1.19 (0.75 - 1.74) | 151 (119- 191) | 2.45 (2.41 - 2.50) | 573 (449 - 711) | 1.92 (1.52 - 2.35) | 1972 (1619 - 2401) | 4.01 (3.33 - 4.80) | 150 (119- 191) | 2.45 (2.41 - 2.50) |
| Central African Republic | 12 (7 - 19) | 0.54 (0.34 - 0.83) | 91 (52 - 146) | 1.83 (1.06 - 2.95) | 252 (147- 441) | 4.30 (3.97 - 4.77) | 43 (33 - 57) | 1.83 (1.42 - 2.30) | 307 (218 - 434) | 6.17 (4.39 - 8.67) | 252 (147- 441) | 4.30 (3.97 - 4.77) |
| Chad | 28 (17 - 42) | 0.54 (0.34 - 0.83) | 297 (164 - 507) | 1.96 (1.07 - 3.35) | 254 (120- 454) | 4.43 (4.19 - 4.77) | 96 (74 - 128) | 1.83 (1.42 - 2.31) | 1004 (627 - 1522) | 6.60 (4.18 - 9.99) | 254 (120- 454) | 4.43 (4.19 - 4.77) |
| Chile | 89 (56 - 128) | 0.74 (0.47 - 1.06) | 287 (184 - 415) | 1.42 (0.91 - 2.05) | 126 (94- 164) | 2.09 (1.87 - 2.24) | 303 (255 - 367) | 2.51 (2.12 - 3.01) | 970 (802 - 1152) | 4.79 (3.96 - 5.66) | 126 (94- 164) | 2.09 (1.87 - 2.24) |
| China | 1894 (1138 - 2960) | 0.17 (0.11 - 0.26) | 2778 (1697 - 4245) | 0.18 (0.11 - 0.29) | 21 (3- 46) | 0.26 (0.22 - 0.30) | 6398 (4668 - 8660) | 0.58 (0.43 - 0.75) | 9386 (6966 - 12260) | 0.62 (0.47 - 0.80) | 21 (3- 46) | 0.26 (0.22 - 0.30) |
| Colombia | 339 (209 - 506) | 1.15 (0.75 - 1.68) | 1179 (731 - 1735) | 2.38 (1.47 - 3.49) | 130 (82- 192) | 2.09 (1.53 - 2.35) | 1146 (905 - 1465) | 3.90 (3.15 - 4.81) | 3982 (3145 - 4903) | 8.03 (6.35 - 9.90) | 130 (82- 192) | 2.09 (1.53 - 2.35) |
| Comoros | 1 (1 - 2) | 0.45 (0.29 - 0.68) | 18 (11 - 27) | 2.62 (1.55 - 3.84) | 527 (344- 775) | 5.25 (3.76 - 5.81) | 6 (4 - 8) | 1.52 (1.19 - 1.89) | 63 (47 - 83) | 8.86 (6.71 - 11.60) | 528 (341- 775) | 5.25 (3.76 - 5.81) |
| Congo | 11 (6 - 17) | 0.54 (0.34 - 0.83) | 104 (63 - 153) | 2.06 (1.27 - 3.01) | 310 (199- 465) | 4.40 (4.40 - 4.41) | 38 (29 - 51) | 1.83 (1.42 - 2.30) | 353 (270 - 451) | 6.95 (5.35 - 8.79) | 310 (199- 465) | 4.40 (4.40 - 4.41) |
| Cook Islands | 0 (0 - 0) | 0.37 (0.23 - 0.58) | 0 (0 - 0) | 0.69 (0.43 - 1.01) | 108 (64- 173) | 1.79 (1.34 - 1.98) | 0 (0 - 0) | 1.26 (0.98 - 1.59) | 0 (0 - 0) | 2.35 (1.82 - 2.98) | 108 (64- 173) | 1.79 (1.34 - 1.98) |
| Costa Rica | 32 (19 - 48) | 1.16 (0.75 - 1.68) | 109 (69 - 167) | 2.25 (1.43 - 3.44) | 115 (61- 186) | 1.84 (1.21 - 2.11) | 109 (86 - 140) | 3.90 (3.16 - 4.82) | 368 (279 - 469) | 7.61 (5.79 - 9.80) | 115 (61- 186) | 1.84 (1.21 - 2.11) |
| C?te d'Ivoire (Ivory Coast) | 21 (13 - 32) | 0.55 (0.35 - 0.84) | 92 (58 - 135) | 2.12 (1.25 - 3.23) | 315 (199- 493) | 4.60 (4.46 - 4.79) | 72 (56 - 92) | 1.85 (1.43 - 2.32) | 313 (248 - 405) | 7.18 (5.19 - 9.17) | 315 (199- 493) | 4.60 (4.46 - 4.79) |
| Croatia | 66 (41 - 99) | 0.42 (0.26 - 0.63) | 115 (74 - 170) | 1.94 (1.21 - 2.89) | 399 (283- 611) | 4.32 (2.82 - 4.95) | 223 (176 - 278) | 1.41 (1.10 - 1.79) | 391 (318 - 474) | 6.56 (5.15 - 8.50) | 399 (283- 611) | 4.32 (2.82 - 4.95) |
| Cuba | 4 (2 - 6) | 0.63 (0.41 - 0.93) | 13 (8 - 19) | 0.91 (0.58 - 1.34) | 69 (44- 101) | 0.98 (0.45 - 1.19) | 14 (11 - 17) | 2.13 (1.69 - 2.64) | 44 (37 - 53) | 3.07 (2.52 - 3.73) | 69 (44- 101) | 0.98 (0.45 - 1.19) |
| Cyprus | 45 (29 - 68) | 0.52 (0.33 - 0.79) | 317 (195 - 472) | 0.86 (0.56 - 1.23) | 81 (62- 107) | 1.37 (0.76 - 1.61) | 155 (120 - 196) | 1.76 (1.38 - 2.21) | 1081 (751 - 1370) | 2.91 (2.44 - 3.45) | 81 (62- 107) | 1.37 (0.76 - 1.61) |
| Czechia | 56 (34 - 86) | 0.42 (0.27 - 0.63) | 536 (316 - 823) | 2.68 (1.63 - 4.01) | 569 (353- 851) | 5.27 (3.67 - 5.98) | 190 (145 - 259) | 1.41 (1.10 - 1.79) | 1811 (1290 - 2321) | 9.09 (6.31 - 11.62) | 576 (359- 850) | 5.27 (3.67 - 5.98) |
| Denmark | 47 (28 - 72) | 0.27 (0.17 - 0.39) | 68 (42 - 102) | 0.72 (0.46 - 1.06) | 170 (129- 225) | 3.07 (2.67 - 3.31) | 159 (119 - 208) | 0.91 (0.74 - 1.14) | 230 (172 - 294) | 2.42 (1.98 - 2.95) | 170 (129- 225) | 3.07 (2.67 - 3.31) |
| Djibouti | 179 (109 - 271) | 0.45 (0.29 - 0.68) | 2222 (1292 - 3305) | 1.76 (1.00 - 2.83) | 322 (172- 584) | 4.66 (4.48 - 4.89) | 604 (463 - 809) | 1.53 (1.19 - 1.91) | 7523 (4984 - 9510) | 5.95 (3.74 - 8.87) | 323 (172- 584) | 4.66 (4.48 - 4.89) |
| Dominica | 15 (9 - 22) | 0.62 (0.40 - 0.93) | 48 (30 - 71) | 0.85 (0.54 - 1.24) | 51 (30- 80) | 0.31 (0.01 - 0.61) | 53 (43 - 66) | 2.10 (1.67 - 2.62) | 164 (132 - 198) | 2.87 (2.30 - 3.58) | 51 (30- 80) | 0.31 (0.01 - 0.61) |
| Dominican Republic | 1 (1 - 2) | 0.63 (0.41 - 0.93) | 21 (12 - 33) | 1.45 (0.80 - 2.31) | 157 (63- 268) | 2.68 (2.53 - 3.05) | 5 (4 - 7) | 2.12 (1.69 - 2.64) | 71 (44 - 107) | 4.89 (3.09 - 6.69) | 158 (63- 268) | 2.68 (2.53 - 3.05) |
| DR Congo | 0 (0 - 0) | 0.54 (0.35 - 0.83) | 0 (0 - 0) | 2.72 (1.59 - 4.02) | 426 (237- 634) | 5.38 (5.33 - 5.45) | 1 (1 - 1) | 1.84 (1.43 - 2.31) | 2 (1 - 2) | 9.22 (6.10 - 11.67) | 426 (238- 634) | 5.38 (5.33 - 5.45) |
| Ecuador | 39 (24 - 60) | 0.83 (0.54 - 1.17) | 157 (86 - 251) | 2.52 (1.54 - 3.81) | 270 (170- 389) | 3.63 (3.56 - 3.69) | 134 (104 - 175) | 2.82 (2.42 - 3.34) | 533 (332 - 730) | 8.51 (6.36 - 10.56) | 270 (170- 389) | 3.63 (3.56 - 3.69) |
| Egypt | 67 (43 - 95) | 0.47 (0.30 - 0.73) | 451 (275 - 682) | 2.16 (0.68 - 3.63) | 400 (71- 696) | 5.24 (5.00 - 5.55) | 227 (194 - 277) | 1.60 (1.24 - 2.02) | 1523 (1138 - 1895) | 7.29 (2.59 - 10.71) | 401 (71- 696) | 5.24 (5.00 - 5.55) |
| El Salvador | 222 (138 - 349) | 1.03 (0.66 - 1.50) | 2128 (662 - 3578) | 1.87 (1.14 - 2.79) | 95 (55- 151) | 1.76 (1.42 - 1.93) | 752 (576 - 986) | 3.49 (2.84 - 4.33) | 7190 (2539 - 10619) | 6.31 (4.91 - 7.83) | 95 (55- 151) | 1.76 (1.42 - 1.93) |
| Equatorial Guinea | 50 (31 - 74) | 0.54 (0.34 - 0.82) | 118 (73 - 178) | 1.92 (1.14 - 2.88) | 272 (159- 412) | 4.77 (4.14 - 5.66) | 169 (132 - 215) | 1.82 (1.42 - 2.29) | 401 (312 - 499) | 6.48 (4.91 - 8.22) | 272 (159- 412) | 4.77 (4.14 - 5.66) |
| Eritrea | 2 (1 - 3) | 0.45 (0.28 - 0.68) | 26 (15 - 39) | 1.01 (0.64 - 1.53) | 135 (82- 223) | 2.17 (1.13 - 2.59) | 6 (5 - 8) | 1.50 (1.17 - 1.89) | 89 (66 - 115) | 3.42 (2.57 - 4.48) | 135 (82- 223) | 2.17 (1.13 - 2.59) |
| Estonia | 13 (8 - 21) | 0.50 (0.31 - 0.76) | 61 (38 - 94) | 1.24 (0.77 - 2.01) | 164 (111- 267) | 2.34 (1.13 - 2.84) | 45 (34 - 60) | 1.68 (1.31 - 2.14) | 208 (155 - 274) | 4.19 (3.28 - 5.59) | 164 (111- 267) | 2.34 (1.13 - 2.84) |
| Eswatini (Swaziland) | 8 (5 - 12) | 0.54 (0.34 - 0.82) | 18 (11 - 29) | 2.31 (1.15 - 3.99) | 378 (176- 704) | 3.95 (2.93 - 4.51) | 27 (21 - 35) | 1.82 (1.41 - 2.29) | 61 (49 - 81) | 7.81 (4.65 - 12.16) | 379 (176- 704) | 3.95 (2.93 - 4.51) |
| Ethiopia | 3 (2 - 5) | 0.50 (0.31 - 0.76) | 25 (12 - 44) | 2.92 (1.84 - 4.28) | 493 (339- 729) | 5.34 (4.48 - 5.76) | 12 (9 - 17) | 1.69 (1.33 - 2.13) | 86 (50 - 133) | 9.92 (7.95 - 11.88) | 495 (340- 729) | 5.34 (4.48 - 5.76) |
| Fiji | 229 (141 - 355) | 0.37 (0.23 - 0.58) | 2934 (1831 - 4302) | 0.88 (0.53 - 1.35) | 160 (72- 268) | 2.24 (0.98 - 2.72) | 774 (595 - 1044) | 1.26 (0.98 - 1.59) | 9933 (7883 - 11991) | 2.98 (1.96 - 3.96) | 160 (72- 268) | 2.24 (0.98 - 2.72) |
| Finland | 2 (1 - 3) | 0.32 (0.21 - 0.46) | 8 (4 - 12) | 0.54 (0.35 - 0.77) | 76 (56- 104) | 1.55 (1.30 - 1.68) | 8 (6 - 11) | 1.09 (0.89 - 1.35) | 27 (17 - 36) | 1.82 (1.54 - 2.16) | 76 (56- 104) | 1.55 (1.30 - 1.68) |
| France | 17 (11 - 25) | 0.53 (0.34 - 0.81) | 34 (22 - 48) | 1.03 (0.65 - 1.53) | 89 (49- 155) | 2.01 (1.69 - 2.24) | 59 (48 - 73) | 1.80 (1.41 - 2.25) | 116 (99 - 138) | 3.48 (2.76 - 4.70) | 89 (49- 155) | 2.01 (1.69 - 2.24) |
| Gabon | 347 (222 - 516) | 0.54 (0.34 - 0.83) | 754 (473 - 1133) | 2.00 (0.95 - 3.17) | 287 (124- 494) | 4.40 (4.28 - 4.56) | 1172 (911 - 1473) | 1.83 (1.42 - 2.30) | 2550 (2053 - 3395) | 6.73 (3.91 - 9.07) | 286 (124- 494) | 4.40 (4.28 - 4.56) |
| Gambia | 4 (2 - 7) | 0.55 (0.35 - 0.83) | 34 (16 - 54) | 2.54 (1.44 - 4.00) | 399 (229- 641) | 4.90 (4.61 - 5.04) | 16 (12 - 21) | 1.84 (1.43 - 2.32) | 115 (67 - 156) | 8.57 (5.70 - 11.41) | 399 (229- 641) | 4.90 (4.61 - 5.04) |
| Georgia | 4 (2 - 6) | 0.54 (0.35 - 0.81) | 55 (31 - 87) | 2.65 (1.23 - 4.22) | 415 (163- 673) | 3.82 (1.94 - 4.75) | 15 (11 - 20) | 1.83 (1.45 - 2.30) | 186 (123 - 248) | 9.02 (4.78 - 12.08) | 420 (173- 671) | 3.82 (1.94 - 4.75) |
| Germany | 31 (19 - 46) | 0.44 (0.27 - 0.65) | 104 (48 - 167) | 0.98 (0.64 - 1.42) | 149 (107- 212) | 2.42 (1.91 - 2.87) | 104 (82 - 130) | 1.47 (1.14 - 1.85) | 356 (188 - 478) | 3.32 (2.81 - 3.89) | 149 (107- 212) | 2.42 (1.91 - 2.87) |
| Ghana | 398 (248 - 606) | 0.54 (0.35 - 0.83) | 1059 (698 - 1552) | 2.11 (1.22 - 3.17) | 315 (179- 486) | 4.31 (4.01 - 4.44) | 1346 (1019 - 1701) | 1.84 (1.43 - 2.31) | 3579 (3017 - 4253) | 7.14 (4.96 - 9.10) | 315 (179- 486) | 4.31 (4.01 - 4.44) |
| Greece | 70 (43 - 107) | 0.32 (0.20 - 0.46) | 671 (387 - 999) | 0.97 (0.64 - 1.39) | 243 (182- 315) | 3.47 (2.77 - 3.93) | 238 (184 - 320) | 1.07 (0.84 - 1.37) | 2267 (1555 - 2902) | 3.29 (2.77 - 3.90) | 243 (182- 315) | 3.47 (2.77 - 3.93) |
| Greenland | 36 (22 - 53) | 0.99 (0.63 - 1.48) | 122 (79 - 178) | 1.07 (0.69 - 1.60) | 43 (27- 61) | 0.21 (0.14 - 0.24) | 123 (96 - 157) | 3.35 (2.66 - 4.15) | 413 (348 - 501) | 3.61 (2.90 - 4.47) | 43 (27- 61) | 0.21 (0.14 - 0.24) |
| Grenada | 0 (0 - 0) | 0.62 (0.40 - 0.93) | 0 (0 - 1) | 0.89 (0.56 - 1.34) | 54 (24- 95) | 0.93 (0.40 - 1.32) | 1 (1 - 2) | 2.11 (1.67 - 2.62) | 2 (1 - 3) | 3.02 (2.36 - 3.83) | 54 (24- 95) | 0.93 (0.40 - 1.32) |
| Guam | 0 (0 - 0) | 0.37 (0.24 - 0.58) | 0 (0 - 1) | 1.44 (0.81 - 2.30) | 329 (188- 588) | 4.16 (3.71 - 4.37) | 1 (1 - 2) | 1.26 (0.99 - 1.59) | 3 (2 - 4) | 4.88 (3.43 - 6.87) | 330 (188- 588) | 4.16 (3.71 - 4.37) |
| Guatemala | 0 (0 - 0) | 1.15 (0.75 - 1.68) | 2 (1 - 3) | 2.84 (1.68 - 4.29) | 150 (79- 241) | 2.76 (2.41 - 3.05) | 1 (1 - 2) | 3.90 (3.15 - 4.82) | 7 (5 - 11) | 9.61 (7.13 - 12.46) | 151 (79- 241) | 2.76 (2.41 - 3.05) |
| Guinea | 91 (54 - 139) | 0.54 (0.35 - 0.83) | 431 (253 - 654) | 2.50 (1.47 - 3.93) | 362 (206- 551) | 5.13 (5.05 - 5.24) | 309 (234 - 414) | 1.84 (1.43 - 2.31) | 1459 (1069 - 1891) | 8.46 (5.82 - 11.26) | 362 (204- 551) | 5.13 (5.05 - 5.24) |
| Guinea-Bissau | 29 (17 - 43) | 0.54 (0.34 - 0.83) | 301 (174 - 481) | 1.78 (0.99 - 2.87) | 242 (126- 457) | 4.46 (3.85 - 5.24) | 98 (76 - 129) | 1.84 (1.42 - 2.31) | 1016 (692 - 1363) | 6.01 (4.12 - 8.78) | 242 (126- 457) | 4.46 (3.85 - 5.24) |
| Guyana | 4 (2 - 7) | 0.63 (0.40 - 0.93) | 32 (17 - 53) | 1.59 (0.84 - 2.65) | 184 (82- 325) | 2.44 (1.64 - 2.85) | 15 (12 - 21) | 2.12 (1.69 - 2.64) | 111 (74 - 162) | 5.36 (3.25 - 7.64) | 183 (82- 325) | 2.44 (1.64 - 2.85) |
| Haiti | 4 (2 - 6) | 0.63 (0.40 - 0.93) | 11 (6 - 20) | 1.19 (0.63 - 1.91) | 97 (24- 188) | 2.07 (2.04 - 2.14) | 14 (11 - 18) | 2.11 (1.68 - 2.63) | 40 (24 - 57) | 4.01 (2.43 - 5.72) | 97 (24- 188) | 2.07 (2.04 - 2.14) |
| Honduras | 35 (22 - 53) | 1.01 (0.64 - 1.50) | 142 (75 - 231) | 3.18 (1.95 - 4.80) | 231 (144- 359) | 3.50 (2.97 - 3.89) | 120 (94 - 158) | 3.42 (2.76 - 4.26) | 481 (287 - 695) | 10.74 (8.15 - 13.47) | 231 (144- 359) | 3.50 (2.97 - 3.89) |
| Hungary | 43 (26 - 65) | 0.42 (0.27 - 0.63) | 306 (188 - 461) | 2.00 (1.19 - 3.09) | 394 (247- 592) | 4.22 (2.50 - 4.94) | 145 (112 - 194) | 1.41 (1.10 - 1.79) | 1036 (773 - 1310) | 6.74 (4.67 - 8.87) | 394 (246- 591) | 4.22 (2.50 - 4.94) |
| Iceland | 47 (29 - 70) | 0.52 (0.33 - 0.80) | 215 (129 - 334) | 0.66 (0.43 - 0.98) | 43 (32- 56) | 0.74 (0.68 - 0.76) | 159 (124 - 202) | 1.76 (1.38 - 2.21) | 726 (501 - 952) | 2.23 (1.81 - 2.71) | 43 (32- 56) | 0.74 (0.68 - 0.76) |
| India | 1 (0 - 2) | 0.74 (0.47 - 1.12) | 2 (1 - 3) | 2.53 (1.61 - 3.72) | 285 (200- 401) | 3.73 (3.11 - 4.01) | 4 (3 - 5) | 2.48 (1.98 - 3.09) | 9 (7 - 11) | 8.53 (6.95 - 10.19) | 285 (200- 401) | 3.73 (3.11 - 4.01) |
| Indonesia | 5592 (3462 - 8547) | 0.59 (0.37 - 0.89) | 35665 (22496 - 52522) | 1.90 (1.19 - 2.75) | 257 (180- 373) | 3.33 (2.25 - 3.79) | 18878 (14683 - 24189) | 1.98 (1.53 - 2.49) | 120438 (98025 - 144220) | 6.43 (5.24 - 7.69) | 257 (180- 373) | 3.33 (2.25 - 3.79) |
| Iran | 984 (613 - 1520) | 0.41 (0.25 - 0.62) | 5294 (3292 - 7640) | 1.98 (1.23 - 2.83) | 467 (320- 651) | 5.06 (4.66 - 5.39) | 3322 (2507 - 4348) | 1.38 (1.07 - 1.75) | 17890 (14541 - 21586) | 6.70 (5.38 - 8.08) | 468 (320- 651) | 5.06 (4.66 - 5.39) |
| Iraq | 201 (122 - 313) | 0.47 (0.30 - 0.73) | 1708 (1051 - 2449) | 3.30 (1.84 - 5.04) | 706 (409- 1077) | 6.55 (6.46 - 6.69) | 680 (518 - 906) | 1.60 (1.24 - 2.02) | 5772 (4656 - 7013) | 11.24 (7.59 - 14.64) | 710 (401- 1069) | 6.55 (6.46 - 6.69) |
| Ireland | 71 (44 - 110) | 0.52 (0.33 - 0.79) | 1286 (728 - 1946) | 1.01 (0.64 - 1.52) | 108 (75- 165) | 1.97 (1.66 - 2.12) | 240 (183 - 321) | 1.76 (1.38 - 2.21) | 4362 (2922 - 5677) | 3.41 (2.70 - 4.33) | 108 (75- 165) | 1.97 (1.66 - 2.12) |
| Israel | 19 (12 - 29) | 0.52 (0.33 - 0.79) | 55 (35 - 83) | 1.15 (0.74 - 1.66) | 133 (97- 184) | 2.33 (1.80 - 2.56) | 65 (51 - 82) | 1.76 (1.37 - 2.20) | 187 (150 - 237) | 3.88 (3.18 - 4.68) | 133 (97- 184) | 2.33 (1.80 - 2.56) |
| Italy | 25 (16 - 38) | 0.66 (0.43 - 0.97) | 113 (73 - 163) | 1.26 (0.82 - 1.78) | 104 (75- 140) | 1.94 (1.61 - 2.10) | 85 (67 - 109) | 2.23 (1.82 - 2.70) | 384 (318 - 462) | 4.25 (3.59 - 5.00) | 104 (75- 140) | 1.94 (1.61 - 2.10) |
| Jamaica | 454 (293 - 673) | 0.63 (0.40 - 0.93) | 975 (638 - 1389) | 1.05 (0.62 - 1.60) | 84 (41- 145) | 1.40 (0.71 - 2.24) | 1536 (1224 - 1885) | 2.11 (1.68 - 2.63) | 3296 (2812 - 3812) | 3.56 (2.59 - 4.56) | 84 (41- 145) | 1.40 (0.71 - 2.24) |
| Japan | 13 (8 - 20) | 1.63 (1.01 - 2.45) | 30 (17 - 45) | 1.93 (1.26 - 2.83) | 16 (4- 32) | 0.50 (0.42 - 0.56) | 46 (37 - 60) | 5.49 (4.42 - 6.86) | 101 (73 - 129) | 6.53 (5.47 - 7.91) | 16 (4- 32) | 0.50 (0.42 - 0.56) |
| Jordan | 2042 (1309 - 3021) | 0.47 (0.30 - 0.73) | 2398 (1586 - 3443) | 2.37 (1.32 - 3.72) | 486 (282- 771) | 4.23 (2.43 - 5.01) | 6896 (5493 - 8510) | 1.60 (1.24 - 2.02) | 8102 (6882 - 9477) | 8.05 (5.60 - 10.35) | 488 (289- 778) | 4.23 (2.43 - 5.01) |
| Kazakhstan | 14 (8 - 21) | 0.54 (0.35 - 0.81) | 273 (155 - 421) | 1.76 (0.92 - 3.06) | 243 (107- 450) | 3.46 (2.89 - 3.76) | 47 (36 - 64) | 1.83 (1.44 - 2.29) | 927 (646 - 1197) | 5.95 (3.58 - 9.11) | 243 (107- 450) | 3.46 (2.89 - 3.76) |
| Kenya | 84 (53 - 128) | 0.52 (0.33 - 0.78) | 333 (173 - 579) | 2.94 (1.85 - 4.33) | 508 (356- 730) | 5.41 (4.76 - 5.71) | 284 (220 - 359) | 1.75 (1.37 - 2.19) | 1127 (676 - 1725) | 9.99 (7.99 - 12.02) | 511 (356- 728) | 5.41 (4.76 - 5.71) |
| Kiribati | 106 (65 - 163) | 0.37 (0.23 - 0.58) | 1405 (882 - 2082) | 0.38 (0.24 - 0.59) | 6 (1- 13) | 0.07 (0.03 - 0.09) | 360 (275 - 486) | 1.26 (0.98 - 1.58) | 4765 (3733 - 5780) | 1.29 (1.01 - 1.64) | 6 (1- 13) | 0.07 (0.03 - 0.09) |
| Kuwait | 0 (0 - 0) | 0.51 (0.33 - 0.75) | 0 (0 - 0) | 1.61 (0.89 - 2.73) | 271 (139- 497) | 3.57 (3.22 - 3.74) | 0 (0 - 1) | 1.71 (1.34 - 2.13) | 1 (1 - 1) | 5.44 (3.57 - 8.39) | 271 (139- 497) | 3.57 (3.22 - 3.74) |
| Kyrgyzstan | 7 (4 - 11) | 0.54 (0.35 - 0.81) | 73 (39 - 125) | 2.45 (1.45 - 3.89) | 373 (222- 611) | 5.06 (4.98 - 5.16) | 24 (18 - 32) | 1.83 (1.45 - 2.29) | 248 (159 - 384) | 8.28 (5.84 - 11.52) | 375 (221- 609) | 5.06 (4.98 - 5.16) |
| Laos | 22 (13 - 33) | 0.53 (0.33 - 0.80) | 161 (95 - 257) | 0.67 (0.43 - 0.99) | 38 (17- 76) | 0.62 (0.29 - 0.75) | 74 (57 - 95) | 1.78 (1.41 - 2.24) | 547 (382 - 761) | 2.26 (1.72 - 2.94) | 38 (17- 76) | 0.62 (0.29 - 0.75) |
| Latvia | 19 (11 - 29) | 0.50 (0.31 - 0.76) | 46 (29 - 69) | 1.79 (0.92 - 3.51) | 281 (127- 550) | 0.37 (-0.25 - 1.01) | 64 (50 - 83) | 1.68 (1.31 - 2.14) | 157 (118 - 207) | 6.02 (3.67 - 10.12) | 279 (127- 550) | 0.37 (-0.25 - 1.01) |
| Lebanon | 14 (8 - 21) | 0.47 (0.30 - 0.73) | 37 (19 - 74) | 2.78 (1.59 - 4.55) | 552 (321- 881) | 5.35 (4.04 - 5.81) | 47 (37 - 59) | 1.60 (1.24 - 2.02) | 127 (78 - 214) | 9.44 (6.44 - 12.79) | 555 (329- 886) | 5.35 (4.04 - 5.81) |
| Lesotho | 13 (8 - 19) | 0.53 (0.34 - 0.81) | 157 (89 - 258) | 2.47 (1.26 - 4.26) | 391 (177- 738) | 3.85 (2.26 - 4.64) | 44 (33 - 56) | 1.80 (1.40 - 2.27) | 534 (363 - 723) | 8.37 (4.50 - 12.86) | 393 (177- 738) | 3.85 (2.26 - 4.64) |
| Liberia | 7 (4 - 11) | 0.55 (0.35 - 0.83) | 44 (22 - 76) | 1.85 (1.10 - 2.81) | 252 (142- 408) | 4.24 (4.00 - 4.57) | 24 (19 - 33) | 1.85 (1.43 - 2.32) | 150 (80 - 232) | 6.24 (4.33 - 8.27) | 254 (142- 408) | 4.24 (4.00 - 4.57) |
| Libya | 11 (7 - 17) | 0.37 (0.24 - 0.54) | 91 (54 - 138) | 2.78 (1.52 - 4.17) | 810 (471- 1192) | 6.10 (4.93 - 6.64) | 39 (30 - 52) | 1.26 (1.03 - 1.54) | 310 (214 - 416) | 9.44 (6.29 - 12.27) | 811 (476- 1185) | 6.10 (4.93 - 6.64) |
| Lithuania | 12 (8 - 19) | 0.50 (0.31 - 0.76) | 189 (103 - 284) | 2.28 (1.23 - 3.81) | 394 (202- 677) | 3.72 (1.78 - 4.61) | 43 (34 - 55) | 1.68 (1.31 - 2.15) | 643 (427 - 839) | 7.70 (4.80 - 11.47) | 394 (204- 679) | 3.72 (1.78 - 4.61) |
| Luxembourg | 19 (11 - 28) | 0.52 (0.33 - 0.79) | 70 (37 - 116) | 1.18 (0.76 - 1.71) | 119 (82- 166) | 2.42 (1.93 - 2.64) | 64 (50 - 81) | 1.76 (1.37 - 2.20) | 236 (147 - 352) | 3.98 (3.24 - 4.81) | 119 (82- 166) | 2.42 (1.93 - 2.64) |
| Madagascar | 2 (1 - 3) | 0.45 (0.29 - 0.68) | 8 (5 - 12) | 2.60 (1.59 - 3.97) | 495 (331- 758) | 5.71 (5.53 - 5.79) | 7 (5 - 9) | 1.52 (1.19 - 1.90) | 28 (23 - 34) | 8.80 (6.58 - 11.36) | 496 (331- 758) | 5.71 (5.53 - 5.79) |
| Malawi | 48 (29 - 75) | 0.45 (0.29 - 0.68) | 686 (417 - 1049) | 2.69 (1.57 - 4.04) | 523 (322- 775) | 5.12 (3.48 - 5.80) | 162 (124 - 212) | 1.52 (1.18 - 1.89) | 2319 (1702 - 3011) | 9.10 (6.32 - 11.35) | 524 (322- 775) | 5.12 (3.48 - 5.80) |
| Malaysia | 39 (23 - 61) | 0.53 (0.33 - 0.80) | 482 (279 - 724) | 1.05 (0.65 - 1.52) | 122 (73- 195) | 1.82 (0.88 - 2.18) | 131 (100 - 173) | 1.78 (1.41 - 2.24) | 1631 (1131 - 2057) | 3.55 (2.74 - 4.45) | 122 (73- 195) | 1.82 (0.88 - 2.18) |
| Maldives | 83 (51 - 129) | 0.53 (0.34 - 0.80) | 333 (206 - 488) | 1.35 (0.84 - 2.29) | 197 (121- 361) | 2.57 (1.84 - 2.92) | 282 (219 - 367) | 1.79 (1.42 - 2.26) | 1128 (876 - 1424) | 4.56 (3.49 - 6.95) | 198 (121- 361) | 2.57 (1.84 - 2.92) |
| Mali | 0 (0 - 1) | 0.54 (0.35 - 0.83) | 6 (4 - 11) | 2.52 (1.38 - 3.77) | 364 (190- 587) | 5.08 (5.06 - 5.11) | 3 (2 - 4) | 1.84 (1.43 - 2.31) | 23 (17 - 35) | 8.51 (5.52 - 11.03) | 364 (190- 587) | 5.08 (5.06 - 5.11) |
| Malta | 41 (25 - 61) | 0.52 (0.33 - 0.79) | 531 (290 - 797) | 1.00 (0.65 - 1.44) | 112 (84- 156) | 1.84 (1.17 - 2.10) | 138 (107 - 184) | 1.76 (1.37 - 2.21) | 1794 (1154 - 2336) | 3.37 (2.80 - 4.02) | 112 (84- 156) | 1.84 (1.17 - 2.10) |
| Marshall Islands | 2 (1 - 3) | 0.37 (0.23 - 0.58) | 5 (3 - 7) | 0.41 (0.25 - 0.61) | 14 (-2- 35) | 0.23 (0.13 - 0.26) | 6 (5 - 8) | 1.26 (0.98 - 1.59) | 17 (14 - 21) | 1.37 (1.05 - 1.74) | 14 (-2- 35) | 0.23 (0.13 - 0.26) |
| Mauritania | 0 (0 - 0) | 0.54 (0.35 - 0.83) | 0 (0 - 0) | 2.22 (1.22 - 3.85) | 325 (172- 582) | 4.36 (3.95 - 4.56) | 0 (0 - 0) | 1.84 (1.43 - 2.31) | 0 (0 - 0) | 7.49 (4.84 - 11.20) | 324 (172- 582) | 4.36 (3.95 - 4.56) |
| Mauritius | 9 (5 - 14) | 0.53 (0.33 - 0.80) | 89 (48 - 154) | 0.65 (0.41 - 0.94) | 42 (23- 67) | 0.56 (0.35 - 0.65) | 33 (25 - 44) | 1.78 (1.41 - 2.24) | 300 (193 - 448) | 2.18 (1.76 - 2.72) | 42 (23- 67) | 0.56 (0.35 - 0.65) |
| Mexico | 5 (3 - 8) | 1.40 (0.91 - 2.04) | 8 (5 - 12) | 3.14 (2.03 - 4.54) | 147 (102- 206) | 2.49 (2.20 - 2.62) | 18 (14 - 23) | 4.73 (3.87 - 5.76) | 30 (23 - 37) | 10.60 (8.88 - 12.44) | 147 (102- 206) | 2.49 (2.20 - 2.62) |
| Micronesia | 1082 (662 - 1615) | 0.37 (0.23 - 0.58) | 4048 (2614 - 5855) | 0.37 (0.23 - 0.58) | 4 (-5- 16) | 0.00 (-0.01 - 0.01) | 3655 (2926 - 4625) | 1.26 (0.98 - 1.59) | 13678 (11451 - 16050) | 1.26 (0.98 - 1.59) | 5 (-5- 16) | 0.00 (-0.01 - 0.01) |
| Moldova | 0 (0 - 0) | 0.50 (0.31 - 0.76) | 0 (0 - 0) | 2.60 (1.43 - 4.27) | 471 (249- 742) | 5.04 (4.39 - 5.36) | 1 (0 - 1) | 1.68 (1.31 - 2.15) | 1 (0 - 1) | 8.81 (5.49 - 12.12) | 474 (245- 758) | 5.04 (4.39 - 5.36) |
| Monaco | 0 (0 - 0) | 0.52 (0.33 - 0.79) | 0 (0 - 0) | 1.02 (0.67 - 1.47) | 81 (60- 110) | 1.90 (1.19 - 2.18) | 0 (0 - 0) | 1.76 (1.37 - 2.20) | 1 (1 - 1) | 3.45 (2.86 - 4.12) | 81 (60- 110) | 1.90 (1.19 - 2.18) |
| Mongolia | 10 (6 - 15) | 0.54 (0.35 - 0.81) | 45 (28 - 66) | 1.41 (0.88 - 2.07) | 185 (118- 275) | 2.48 (1.08 - 3.01) | 34 (26 - 45) | 1.84 (1.45 - 2.30) | 152 (114 - 188) | 4.74 (3.56 - 5.83) | 185 (118- 275) | 2.48 (1.08 - 3.01) |
| Montenegro | 2 (1 - 4) | 0.43 (0.28 - 0.67) | 20 (12 - 30) | 3.09 (1.96 - 4.56) | 662 (463- 957) | 6.07 (4.48 - 6.78) | 9 (7 - 11) | 1.47 (1.15 - 1.86) | 69 (55 - 83) | 10.49 (8.29 - 12.64) | 667 (479- 940) | 6.07 (4.48 - 6.78) |
| Morocco | 103 (64 - 161) | 0.47 (0.30 - 0.73) | 868 (392 - 1467) | 2.33 (1.05 - 3.93) | 472 (229- 778) | 4.69 (3.94 - 5.09) | 349 (269 - 459) | 1.60 (1.24 - 2.02) | 2920 (1701 - 4054) | 7.84 (4.57 - 10.91) | 469 (229- 780) | 4.69 (3.94 - 5.09) |
| Mozambique | 54 (33 - 84) | 0.45 (0.28 - 0.68) | 848 (503 - 1277) | 3.01 (1.76 - 4.59) | 573 (365- 859) | 5.25 (3.19 - 6.11) | 182 (140 - 239) | 1.51 (1.18 - 1.89) | 2876 (1994 - 3637) | 10.27 (7.17 - 12.92) | 576 (359- 843) | 5.25 (3.19 - 6.11) |
| Myanmar | 192 (117 - 296) | 0.53 (0.33 - 0.80) | 650 (387 - 1002) | 1.16 (0.69 - 1.78) | 142 (77- 234) | 2.11 (1.20 - 2.48) | 649 (506 - 840) | 1.78 (1.41 - 2.24) | 2199 (1553 - 2802) | 3.92 (2.79 - 4.97) | 143 (77- 234) | 2.11 (1.20 - 2.48) |
| Namibia | 6 (4 - 10) | 0.54 (0.34 - 0.83) | 49 (26 - 80) | 2.14 (1.17 - 3.42) | 332 (190- 549) | 3.54 (1.93 - 4.24) | 22 (17 - 30) | 1.83 (1.42 - 2.29) | 168 (108 - 232) | 7.21 (4.74 - 9.92) | 331 (190- 549) | 3.54 (1.93 - 4.24) |
| Nauru | 0 (0 - 0) | 0.38 (0.24 - 0.58) | 0 (0 - 0) | 0.65 (0.41 - 0.95) | 85 (48- 141) | 1.56 (1.16 - 1.74) | 0 (0 - 0) | 1.27 (0.99 - 1.60) | 0 (0 - 0) | 2.18 (1.73 - 2.76) | 85 (48- 141) | 1.56 (1.16 - 1.74) |
| Nepal | 111 (68 - 167) | 0.66 (0.42 - 0.98) | 770 (454 - 1213) | 2.52 (1.50 - 3.95) | 334 (219- 570) | 3.79 (2.72 - 4.31) | 375 (295 - 484) | 2.24 (1.80 - 2.80) | 2599 (1968 - 3617) | 8.52 (6.47 - 11.81) | 333 (219- 570) | 3.79 (2.72 - 4.31) |
| Netherlands | 67 (43 - 102) | 0.41 (0.27 - 0.62) | 235 (148 - 360) | 1.18 (0.73 - 1.79) | 200 (146- 290) | 3.23 (2.87 - 3.42) | 229 (177 - 294) | 1.40 (1.08 - 1.78) | 797 (643 - 1042) | 3.98 (3.18 - 5.22) | 200 (146- 290) | 3.23 (2.87 - 3.42) |
| New Zealand | 23 (15 - 34) | 0.67 (0.43 - 0.96) | 38 (24 - 53) | 0.63 (0.41 - 0.88) | 5 (-3- 14) | -0.22 (-0.23 - -0.21) | 81 (66 - 96) | 2.27 (1.87 - 2.73) | 128 (107 - 151) | 2.12 (1.79 - 2.52) | 5 (-3- 14) | -0.22 (-0.23 - -0.21) |
| Nicaragua | 41 (24 - 63) | 1.15 (0.74 - 1.67) | 162 (103 - 236) | 2.52 (1.61 - 3.67) | 127 (82- 192) | 2.36 (1.94 - 2.60) | 140 (106 - 187) | 3.87 (3.14 - 4.79) | 548 (437 - 662) | 8.52 (6.82 - 10.28) | 128 (82- 192) | 2.36 (1.94 - 2.60) |
| Niger | 37 (22 - 56) | 0.55 (0.35 - 0.83) | 402 (233 - 650) | 1.88 (1.09 - 3.02) | 248 (129- 425) | 4.00 (3.90 - 4.05) | 125 (96 - 169) | 1.84 (1.43 - 2.32) | 1358 (873 - 1940) | 6.33 (4.14 - 8.99) | 248 (129- 425) | 4.00 (3.90 - 4.05) |
| Nigeria | 484 (296 - 747) | 0.61 (0.38 - 0.93) | 5206 (3313 - 7759) | 2.51 (1.60 - 3.70) | 319 (225- 446) | 4.79 (4.68 - 4.95) | 1633 (1273 - 2142) | 2.04 (1.61 - 2.57) | 17590 (14085 - 21150) | 8.49 (6.93 - 10.19) | 319 (225- 447) | 4.79 (4.68 - 4.95) |
| Niue | 0 (0 - 0) | 0.37 (0.23 - 0.58) | 0 (0 - 0) | 0.37 (0.23 - 0.58) | 6 (-3- 15) | 0.00 (0.00 - 0.00) | 0 (0 - 0) | 1.26 (0.98 - 1.59) | 0 (0 - 0) | 1.26 (0.98 - 1.59) | 6 (-3- 15) | 0.00 (0.00 - 0.00) |
| North Korea | 8 (5 - 12) | 0.24 (0.15 - 0.37) | 79 (45 - 121) | 0.25 (0.15 - 0.38) | 13 (3- 23) | 0.08 (0.08 - 0.09) | 28 (21 - 35) | 0.82 (0.63 - 1.05) | 270 (194 - 341) | 0.84 (0.65 - 1.08) | 13 (3- 23) | 0.08 (0.08 - 0.09) |
| North Macedonia | 0 (0 - 0) | 0.42 (0.27 - 0.63) | 0 (0 - 0) | 3.37 (1.99 - 5.11) | 774 (476- 1212) | 6.32 (5.30 - 6.80) | 0 (0 - 0) | 1.42 (1.11 - 1.80) | 0 (0 - 1) | 11.47 (8.21 - 14.48) | 781 (528- 1122) | 6.32 (5.30 - 6.80) |
| Northern Mariana Islands | 40 (26 - 60) | 0.37 (0.24 - 0.58) | 78 (51 - 112) | 0.56 (0.36 - 0.84) | 71 (41- 114) | 1.26 (1.17 - 1.30) | 136 (109 - 170) | 1.27 (0.99 - 1.60) | 263 (222 - 309) | 1.89 (1.53 - 2.32) | 71 (41- 114) | 1.26 (1.17 - 1.30) |
| Norway | 7 (4 - 12) | 0.84 (0.54 - 1.25) | 68 (41 - 108) | 1.23 (0.81 - 1.78) | 51 (34- 73) | 1.12 (0.98 - 1.22) | 25 (19 - 35) | 2.85 (2.27 - 3.49) | 233 (163 - 345) | 4.15 (3.56 - 4.91) | 51 (34- 73) | 1.12 (0.98 - 1.22) |
| Oman | 716 (440 - 1087) | 0.48 (0.31 - 0.73) | 5425 (3359 - 8208) | 1.55 (0.92 - 2.42) | 278 (158- 484) | 3.69 (3.39 - 3.84) | 2418 (1883 - 3107) | 1.61 (1.25 - 2.05) | 18334 (13697 - 23317) | 5.25 (3.66 - 7.65) | 280 (158- 484) | 3.69 (3.39 - 3.84) |
| Pakistan | 0 (0 - 0) | 0.73 (0.47 - 1.11) | 0 (0 - 0) | 2.44 (1.50 - 3.66) | 257 (166- 386) | 3.90 (3.80 - 3.94) | 0 (0 - 0) | 2.47 (1.97 - 3.08) | 0 (0 - 0) | 8.23 (6.17 - 10.39) | 258 (166- 386) | 3.90 (3.80 - 3.94) |
| Palau | 7 (4 - 12) | 0.37 (0.23 - 0.58) | 143 (78 - 227) | 0.38 (0.24 - 0.58) | 12 (-2- 31) | 0.03 (0.02 - 0.03) | 26 (20 - 35) | 1.26 (0.98 - 1.59) | 487 (313 - 642) | 1.27 (0.99 - 1.60) | 12 (-2- 31) | 0.03 (0.02 - 0.03) |
| Palestine | 25 (15 - 37) | 0.47 (0.30 - 0.73) | 97 (63 - 143) | 3.04 (1.62 - 4.79) | 633 (358- 1007) | 5.21 (3.71 - 5.91) | 86 (68 - 109) | 1.60 (1.24 - 2.02) | 330 (264 - 401) | 10.33 (6.66 - 13.70) | 636 (340- 978) | 5.21 (3.71 - 5.91) |
| Panama | 13 (8 - 21) | 1.16 (0.75 - 1.69) | 104 (57 - 177) | 2.27 (1.48 - 3.33) | 112 (73- 165) | 2.09 (1.83 - 2.43) | 45 (34 - 61) | 3.92 (3.17 - 4.84) | 354 (226 - 522) | 7.67 (6.14 - 9.35) | 112 (73- 165) | 2.09 (1.83 - 2.43) |
| Papua New Guinea | 12 (7 - 18) | 0.37 (0.23 - 0.58) | 147 (86 - 233) | 1.04 (0.58 - 1.74) | 202 (95- 377) | 2.49 (0.99 - 3.12) | 40 (31 - 52) | 1.26 (0.98 - 1.59) | 498 (345 - 670) | 3.52 (2.29 - 5.18) | 202 (95- 377) | 2.49 (0.99 - 3.12) |
| Paraguay | 142 (90 - 211) | 0.38 (0.24 - 0.57) | 897 (544 - 1368) | 2.09 (1.24 - 3.30) | 592 (376- 921) | 4.70 (3.02 - 5.43) | 482 (383 - 602) | 1.28 (0.98 - 1.65) | 3035 (2299 - 3942) | 7.05 (4.88 - 9.44) | 591 (376- 921) | 4.70 (3.02 - 5.43) |
| Peru | 328 (204 - 508) | 0.80 (0.52 - 1.18) | 1881 (1199 - 2759) | 2.48 (1.51 - 3.77) | 275 (181- 415) | 3.74 (3.69 - 3.87) | 1107 (839 - 1470) | 2.69 (2.15 - 3.29) | 6357 (5207 - 7505) | 8.39 (6.38 - 10.91) | 276 (181- 415) | 3.74 (3.69 - 3.87) |
| Philippines | 184 (115 - 287) | 0.59 (0.37 - 0.89) | 827 (523 - 1207) | 1.68 (1.08 - 2.47) | 219 (162- 309) | 3.01 (2.31 - 3.31) | 622 (464 - 813) | 1.99 (1.54 - 2.49) | 2794 (2240 - 3372) | 5.68 (4.67 - 6.69) | 219 (162- 309) | 3.01 (2.31 - 3.31) |
| Poland | 59 (37 - 90) | 0.47 (0.29 - 0.73) | 150 (95 - 218) | 1.95 (1.22 - 2.85) | 348 (236- 521) | 4.00 (2.63 - 4.60) | 200 (153 - 254) | 1.59 (1.18 - 2.07) | 507 (410 - 629) | 6.58 (5.21 - 7.98) | 348 (236- 521) | 4.00 (2.63 - 4.60) |
| Portugal | 22 (14 - 33) | 0.52 (0.33 - 0.79) | 31 (19 - 44) | 1.20 (0.76 - 1.73) | 142 (99- 204) | 2.42 (1.70 - 2.72) | 75 (60 - 95) | 1.75 (1.37 - 2.20) | 107 (85 - 131) | 4.07 (3.27 - 5.12) | 142 (99- 204) | 2.42 (1.70 - 2.72) |
| Puerto Rico | 1 (1 - 2) | 0.62 (0.40 - 0.93) | 55 (30 - 82) | 0.85 (0.54 - 1.20) | 55 (26- 93) | 0.56 (0.04 - 1.08) | 6 (4 - 8) | 2.11 (1.68 - 2.63) | 186 (121 - 242) | 2.86 (2.30 - 3.51) | 55 (26- 93) | 0.56 (0.04 - 1.08) |
| Qatar | 778 (480 - 1191) | 0.48 (0.31 - 0.74) | 931 (603 - 1398) | 1.97 (1.07 - 2.96) | 361 (193- 585) | 5.03 (4.66 - 5.54) | 2625 (2053 - 3344) | 1.62 (1.25 - 2.06) | 3143 (2531 - 3878) | 6.66 (4.39 - 8.59) | 361 (193- 585) | 5.03 (4.66 - 5.54) |
| Romania | 22 (13 - 33) | 0.42 (0.27 - 0.63) | 102 (55 - 167) | 2.05 (1.12 - 3.16) | 420 (250- 648) | 4.58 (3.51 - 5.08) | 75 (58 - 95) | 1.42 (1.11 - 1.79) | 348 (220 - 480) | 6.92 (4.71 - 9.27) | 419 (250- 648) | 4.58 (3.51 - 5.08) |
| Russia | 102 (64 - 153) | 0.55 (0.34 - 0.81) | 432 (239 - 664) | 2.63 (1.65 - 3.85) | 405 (301- 555) | 4.79 (4.05 - 5.12) | 346 (270 - 437) | 1.85 (1.45 - 2.32) | 1458 (1004 - 1955) | 8.90 (7.15 - 10.83) | 407 (302- 563) | 4.79 (4.05 - 5.12) |
| Rwanda | 858 (545 - 1285) | 0.45 (0.28 - 0.68) | 4156 (2652 - 6060) | 1.45 (0.85 - 2.28) | 238 (123- 401) | 3.01 (1.44 - 3.65) | 2897 (2261 - 3624) | 1.51 (1.18 - 1.89) | 14091 (11390 - 17007) | 4.88 (3.34 - 6.70) | 239 (123- 401) | 3.01 (1.44 - 3.65) |
| Saint Kitts and Nevis | 28 (17 - 45) | 0.62 (0.40 - 0.93) | 179 (105 - 282) | 0.78 (0.50 - 1.13) | 36 (16- 66) | 0.24 (0.03 - 0.46) | 97 (73 - 128) | 2.11 (1.67 - 2.62) | 607 (415 - 847) | 2.63 (2.10 - 3.21) | 37 (16- 66) | 0.24 (0.03 - 0.46) |
| Saint Lucia | 0 (0 - 0) | 0.62 (0.40 - 0.93) | 0 (0 - 0) | 1.12 (0.64 - 1.73) | 109 (57- 174) | 1.46 (0.55 - 1.92) | 0 (0 - 1) | 2.10 (1.67 - 2.62) | 1 (1 - 2) | 3.78 (2.73 - 4.91) | 109 (57- 174) | 1.46 (0.55 - 1.92) |
| Saint Vincent and the Grenadines | 0 (0 - 1) | 0.62 (0.40 - 0.93) | 2 (1 - 3) | 0.92 (0.58 - 1.36) | 69 (38- 108) | 1.03 (0.51 - 1.47) | 2 (2 - 3) | 2.11 (1.68 - 2.63) | 7 (5 - 9) | 3.11 (2.48 - 3.87) | 69 (38- 108) | 1.03 (0.51 - 1.47) |
| Samoa | 0 (0 - 0) | 0.37 (0.23 - 0.58) | 1 (0 - 1) | 0.38 (0.24 - 0.59) | 5 (0- 12) | 0.05 (0.03 - 0.06) | 2 (1 - 2) | 1.26 (0.98 - 1.59) | 3 (2 - 4) | 1.28 (1.00 - 1.62) | 5 (0- 12) | 0.05 (0.03 - 0.06) |
| San Marino | 0 (0 - 0) | 0.52 (0.33 - 0.80) | 0 (0 - 1) | 1.68 (1.06 - 2.44) | 223 (155- 319) | 3.77 (3.66 - 3.83) | 1 (1 - 2) | 1.76 (1.38 - 2.21) | 2 (1 - 3) | 5.66 (4.49 - 6.91) | 223 (155- 319) | 3.77 (3.66 - 3.83) |
| Sao Tome and Principe | 0 (0 - 0) | 0.54 (0.34 - 0.83) | 0 (0 - 0) | 1.53 (0.94 - 2.27) | 196 (122- 308) | 3.67 (3.33 - 4.17) | 0 (0 - 0) | 1.83 (1.42 - 2.30) | 2 (1 - 2) | 5.16 (3.99 - 6.67) | 197 (122- 308) | 3.67 (3.33 - 4.17) |
| Saudi Arabia | 0 (0 - 0) | 0.48 (0.31 - 0.73) | 3 (1 - 4) | 0.96 (0.55 - 1.52) | 135 (74- 241) | 2.54 (2.29 - 2.86) | 1 (1 - 2) | 1.61 (1.24 - 2.04) | 10 (7 - 13) | 3.24 (2.31 - 4.62) | 135 (74- 241) | 2.54 (2.29 - 2.86) |
| Senegal | 61 (37 - 96) | 0.54 (0.35 - 0.83) | 343 (200 - 546) | 2.77 (1.66 - 4.17) | 444 (284- 675) | 4.94 (4.18 - 5.29) | 207 (157 - 280) | 1.84 (1.43 - 2.31) | 1160 (815 - 1652) | 9.37 (7.00 - 12.16) | 446 (286- 674) | 4.94 (4.18 - 5.29) |
| Serbia | 35 (21 - 54) | 0.37 (0.23 - 0.55) | 405 (240 - 607) | 1.93 (1.17 - 2.96) | 455 (268- 742) | 5.43 (3.91 - 6.22) | 121 (93 - 162) | 1.23 (0.95 - 1.58) | 1372 (1025 - 1794) | 6.53 (4.46 - 8.65) | 455 (269- 743) | 5.43 (3.91 - 6.22) |
| Seychelles | 36 (22 - 55) | 0.53 (0.33 - 0.80) | 189 (114 - 291) | 1.68 (1.10 - 2.49) | 238 (164- 342) | 3.11 (1.48 - 3.73) | 124 (93 - 159) | 1.78 (1.41 - 2.24) | 640 (437 - 847) | 5.67 (4.45 - 6.99) | 238 (164- 342) | 3.11 (1.48 - 3.73) |
| Sierra Leone | 0 (0 - 0) | 0.55 (0.35 - 0.83) | 1 (1 - 2) | 1.31 (0.79 - 1.96) | 147 (90- 243) | 3.30 (2.83 - 3.99) | 1 (0 - 1) | 1.84 (1.43 - 2.32) | 5 (4 - 7) | 4.44 (3.19 - 5.93) | 148 (90- 243) | 3.30 (2.83 - 3.99) |
| Singapore | 19 (12 - 30) | 1.73 (1.09 - 2.59) | 104 (62 - 157) | 1.81 (1.15 - 2.71) | 5 (-5- 17) | 0.16 (0.15 - 0.17) | 66 (51 - 88) | 5.82 (4.72 - 7.27) | 354 (253 - 472) | 6.09 (4.97 - 7.55) | 5 (-5- 17) | 0.16 (0.15 - 0.17) |
| Slovakia | 53 (33 - 82) | 0.42 (0.27 - 0.63) | 105 (68 - 158) | 1.83 (1.10 - 2.85) | 367 (231- 599) | 3.86 (1.98 - 4.63) | 180 (142 - 231) | 1.41 (1.10 - 1.79) | 355 (284 - 441) | 6.18 (4.53 - 9.08) | 367 (231- 599) | 3.86 (1.98 - 4.63) |
| Slovenia | 22 (14 - 34) | 0.42 (0.26 - 0.63) | 109 (66 - 169) | 1.60 (0.86 - 2.91) | 316 (170- 599) | 3.27 (1.74 - 4.02) | 76 (59 - 97) | 1.41 (1.10 - 1.79) | 368 (273 - 541) | 5.40 (3.47 - 8.60) | 315 (170- 599) | 3.27 (1.74 - 4.02) |
| Solomon Islands | 8 (5 - 12) | 0.38 (0.24 - 0.58) | 37 (20 - 68) | 0.39 (0.24 - 0.59) | 6 (-1- 17) | 0.08 (0.04 - 0.09) | 29 (22 - 36) | 1.27 (0.99 - 1.59) | 127 (83 - 205) | 1.30 (1.02 - 1.66) | 6 (-1- 17) | 0.08 (0.04 - 0.09) |
| Somalia | 1 (0 - 1) | 0.45 (0.29 - 0.68) | 2 (1 - 3) | 2.04 (1.20 - 3.07) | 353 (220- 543) | 4.84 (4.47 - 4.98) | 3 (2 - 5) | 1.53 (1.19 - 1.91) | 8 (6 - 10) | 6.89 (5.15 - 8.79) | 354 (220- 543) | 4.84 (4.47 - 4.98) |
| South Africa | 31 (19 - 50) | 0.60 (0.38 - 0.91) | 392 (226 - 595) | 2.25 (1.42 - 3.30) | 313 (225- 440) | 4.02 (3.37 - 4.32) | 107 (80 - 141) | 2.02 (1.60 - 2.55) | 1324 (978 - 1707) | 7.61 (6.11 - 9.12) | 313 (225- 440) | 4.02 (3.37 - 4.32) |
| South Korea | 202 (125 - 316) | 1.73 (1.09 - 2.60) | 1282 (807 - 1861) | 1.77 (1.12 - 2.67) | 3 (-8- 18) | 0.07 (0.06 - 0.08) | 682 (522 - 896) | 5.83 (4.72 - 7.28) | 4334 (3470 - 5215) | 5.97 (4.84 - 7.42) | 3 (-8- 18) | 0.07 (0.06 - 0.08) |
| South Sudan | 23 (14 - 37) | 0.46 (0.29 - 0.69) | 145 (75 - 256) | 1.66 (0.87 - 2.93) | 269 (139- 524) | 4.35 (4.25 - 4.48) | 80 (61 - 105) | 1.54 (1.20 - 1.92) | 489 (310 - 777) | 5.61 (3.56 - 9.00) | 269 (139- 524) | 4.35 (4.25 - 4.48) |
| Spain | 127 (79 - 182) | 0.31 (0.19 - 0.44) | 710 (464 - 1026) | 1.33 (0.86 - 1.92) | 373 (292- 493) | 4.67 (4.28 - 4.93) | 431 (343 - 536) | 1.04 (0.83 - 1.31) | 2398 (1990 - 2882) | 4.48 (3.70 - 5.47) | 373 (292- 493) | 4.67 (4.28 - 4.93) |
| Sri Lanka | 83 (51 - 130) | 0.53 (0.33 - 0.80) | 184 (117 - 271) | 0.80 (0.51 - 1.19) | 69 (43- 108) | 1.10 (0.53 - 1.32) | 283 (220 - 366) | 1.79 (1.41 - 2.25) | 624 (501 - 769) | 2.71 (2.18 - 3.36) | 69 (43- 108) | 1.10 (0.53 - 1.32) |
| Sudan | 78 (48 - 121) | 0.47 (0.30 - 0.73) | 660 (303 - 1078) | 1.70 (0.80 - 2.77) | 288 (98- 519) | 4.54 (4.19 - 5.00) | 264 (203 - 350) | 1.60 (1.24 - 2.02) | 2233 (1117 - 3235) | 5.75 (2.95 - 8.28) | 289 (98- 519) | 4.54 (4.19 - 5.00) |
| Suriname | 2 (1 - 3) | 0.63 (0.41 - 0.93) | 9 (5 - 14) | 1.59 (0.91 - 2.52) | 180 (87- 288) | 2.52 (1.64 - 3.01) | 7 (5 - 9) | 2.12 (1.69 - 2.64) | 31 (21 - 42) | 5.36 (3.59 - 7.12) | 180 (87- 288) | 2.52 (1.64 - 3.01) |
| Sweden | 47 (29 - 69) | 0.48 (0.30 - 0.72) | 169 (110 - 245) | 1.39 (0.91 - 2.02) | 197 (160- 254) | 3.37 (3.18 - 3.49) | 159 (123 - 199) | 1.61 (1.26 - 2.02) | 572 (479 - 683) | 4.70 (3.93 - 5.60) | 197 (160- 254) | 3.37 (3.18 - 3.49) |
| Switzerland | 41 (25 - 62) | 0.52 (0.33 - 0.79) | 109 (70 - 160) | 1.05 (0.67 - 1.53) | 104 (76- 146) | 2.04 (1.53 - 2.26) | 139 (106 - 176) | 1.76 (1.38 - 2.21) | 370 (308 - 450) | 3.55 (2.93 - 4.32) | 104 (76- 146) | 2.04 (1.53 - 2.26) |
| Syria | 48 (30 - 75) | 0.47 (0.30 - 0.73) | 107 (63 - 178) | 0.76 (0.45 - 1.26) | 99 (48- 174) | 1.20 (0.66 - 1.43) | 165 (125 - 220) | 1.60 (1.24 - 2.02) | 362 (254 - 518) | 2.55 (1.79 - 3.66) | 99 (48- 174) | 1.20 (0.66 - 1.43) |
| Taiwan | 47 (29 - 69) | 0.24 (0.15 - 0.36) | 81 (52 - 116) | 0.30 (0.19 - 0.43) | 50 (27- 76) | 0.66 (0.63 - 0.68) | 159 (125 - 201) | 0.82 (0.67 - 1.03) | 277 (231 - 336) | 1.02 (0.84 - 1.24) | 50 (27- 76) | 0.66 (0.63 - 0.68) |
| Tajikistan | 25 (15 - 38) | 0.54 (0.35 - 0.81) | 208 (119 - 323) | 2.19 (1.25 - 3.38) | 331 (195- 515) | 4.18 (3.54 - 4.49) | 86 (65 - 112) | 1.83 (1.45 - 2.30) | 703 (484 - 912) | 7.38 (5.15 - 9.55) | 331 (195- 515) | 4.18 (3.54 - 4.49) |
| Tanzania | 279 (172 - 430) | 0.36 (0.23 - 0.55) | 511 (314 - 773) | 2.20 (1.38 - 3.23) | 497 (322- 721) | 5.67 (4.81 - 6.09) | 941 (732 - 1222) | 1.22 (0.96 - 1.57) | 1727 (1357 - 2195) | 7.44 (5.86 - 9.06) | 498 (322- 721) | 5.67 (4.81 - 6.09) |
| Thailand | 3 (2 - 5) | 0.53 (0.33 - 0.80) | 13 (8 - 20) | 0.69 (0.43 - 1.05) | 56 (28- 92) | 0.70 (0.31 - 0.85) | 11 (9 - 15) | 1.78 (1.41 - 2.24) | 45 (32 - 61) | 2.34 (1.84 - 2.98) | 56 (28- 92) | 0.70 (0.31 - 0.85) |
| Timor-Leste (East Timor) | 16 (10 - 25) | 0.53 (0.33 - 0.80) | 143 (84 - 225) | 1.01 (0.61 - 1.55) | 113 (56- 210) | 1.62 (0.63 - 1.99) | 56 (43 - 77) | 1.79 (1.41 - 2.25) | 484 (340 - 646) | 3.40 (2.50 - 4.49) | 114 (56- 210) | 1.62 (0.63 - 1.99) |
| Togo | 0 (0 - 0) | 0.54 (0.34 - 0.83) | 0 (0 - 0) | 1.86 (1.11 - 2.90) | 272 (162- 426) | 3.48 (2.58 - 3.90) | 0 (0 - 0) | 1.83 (1.42 - 2.31) | 0 (0 - 0) | 6.28 (4.46 - 8.25) | 272 (162- 426) | 3.48 (2.58 - 3.90) |
| Tokelau | 0 (0 - 0) | 0.37 (0.23 - 0.58) | 0 (0 - 0) | 0.37 (0.23 - 0.58) | 5 (-1- 12) | 0.00 (0.00 - 0.00) | 1 (0 - 1) | 1.26 (0.98 - 1.59) | 1 (0 - 1) | 1.26 (0.98 - 1.59) | 5 (-1- 12) | 0.00 (0.00 - 0.00) |
| Tonga | 7 (4 - 10) | 0.37 (0.23 - 0.58) | 15 (9 - 23) | 0.37 (0.23 - 0.58) | 4 (0- 10) | 0.00 (-0.00 - 0.01) | 23 (18 - 30) | 1.26 (0.98 - 1.59) | 52 (40 - 67) | 1.26 (0.98 - 1.59) | 4 (0- 10) | 0.00 (-0.00 - 0.01) |
| Trinidad and Tobago | 34 (21 - 54) | 0.63 (0.40 - 0.93) | 338 (181 - 552) | 1.04 (0.65 - 1.54) | 90 (46- 147) | 1.37 (0.79 - 1.60) | 117 (90 - 153) | 2.12 (1.69 - 2.64) | 1148 (701 - 1546) | 3.51 (2.72 - 4.47) | 90 (46- 147) | 1.37 (0.79 - 1.60) |
| Tunisia | 268 (166 - 404) | 0.47 (0.30 - 0.73) | 1474 (872 - 2428) | 2.75 (1.47 - 4.50) | 587 (303- 947) | 4.61 (2.64 - 5.48) | 907 (703 - 1159) | 1.60 (1.24 - 2.02) | 4980 (3372 - 6985) | 9.34 (5.69 - 12.60) | 590 (322- 944) | 4.61 (2.64 - 5.48) |
| Turkey | 17 (10 - 26) | 0.53 (0.33 - 0.79) | 111 (66 - 180) | 1.69 (1.00 - 2.78) | 277 (152- 466) | 3.62 (3.32 - 3.75) | 59 (45 - 77) | 1.79 (1.40 - 2.23) | 377 (263 - 503) | 5.70 (3.88 - 8.03) | 277 (152- 466) | 3.62 (3.32 - 3.75) |
| Turkmenistan | 0 (0 - 0) | 0.54 (0.35 - 0.81) | 0 (0 - 0) | 2.21 (1.33 - 3.56) | 353 (216- 545) | 4.22 (3.57 - 4.53) | 0 (0 - 0) | 1.83 (1.45 - 2.30) | 0 (0 - 0) | 7.47 (5.28 - 9.92) | 353 (217- 545) | 4.22 (3.57 - 4.53) |
| Tuvalu | 68 (41 - 107) | 0.37 (0.23 - 0.58) | 680 (418 - 1050) | 0.65 (0.42 - 0.95) | 85 (52- 141) | 1.64 (1.23 - 1.81) | 231 (176 - 305) | 1.25 (0.97 - 1.58) | 2296 (1696 - 3025) | 2.21 (1.75 - 2.82) | 85 (52- 141) | 1.64 (1.23 - 1.81) |
| U.S. Virgin Islands | 307 (196 - 462) | 0.62 (0.40 - 0.92) | 1062 (569 - 1831) | 1.13 (0.70 - 1.70) | 119 (76- 188) | 1.75 (1.38 - 2.16) | 1037 (807 - 1291) | 2.10 (1.67 - 2.62) | 3598 (2194 - 5441) | 3.81 (2.95 - 5.12) | 119 (76- 188) | 1.75 (1.38 - 2.16) |
| Uganda | 7 (4 - 11) | 0.45 (0.29 - 0.68) | 171 (92 - 273) | 1.74 (1.08 - 2.64) | 296 (188- 450) | 3.70 (2.19 - 4.32) | 24 (18 - 34) | 1.52 (1.18 - 1.89) | 579 (361 - 848) | 5.89 (4.39 - 7.76) | 296 (188- 450) | 3.70 (2.19 - 4.32) |
| Ukraine | 146 (91 - 224) | 0.55 (0.35 - 0.81) | 832 (545 - 1185) | 2.21 (1.20 - 3.80) | 323 (162- 584) | 3.72 (2.47 - 4.33) | 495 (377 - 642) | 1.85 (1.45 - 2.33) | 2811 (2338 - 3289) | 7.50 (4.57 - 11.30) | 324 (163- 583) | 3.72 (2.47 - 4.33) |
| United Arab Emirates | 87 (53 - 138) | 0.48 (0.31 - 0.74) | 1185 (740 - 1737) | 1.74 (0.96 - 2.77) | 351 (174- 592) | 3.89 (3.29 - 4.17) | 296 (224 - 404) | 1.61 (1.25 - 2.05) | 4005 (3105 - 4929) | 5.89 (3.65 - 8.66) | 351 (174- 592) | 3.89 (3.29 - 4.17) |
| United Kingdom | 2971 (1922 - 4418) | 0.22 (0.14 - 0.34) | 8732 (5751 - 12209) | 1.09 (0.70 - 1.57) | 379 (304- 479) | 5.13 (4.90 - 5.28) | 10033 (8206 - 12264) | 0.74 (0.57 - 0.95) | 29505 (25851 - 33751) | 3.69 (3.04 - 4.37) | 379 (304- 479) | 5.13 (4.90 - 5.28) |
| United States | 0 (0 - 0) | 1.07 (0.69 - 1.59) | 1 (0 - 1) | 2.15 (1.41 - 3.05) | 125 (93- 167) | 2.06 (1.67 - 2.36) | 2 (1 - 2) | 3.62 (2.95 - 4.37) | 3 (2 - 5) | 7.26 (6.27 - 8.27) | 125 (93- 167) | 2.06 (1.67 - 2.36) |
| Uruguay | 33 (21 - 49) | 0.98 (0.63 - 1.46) | 59 (38 - 86) | 1.58 (1.01 - 2.30) | 65 (48- 92) | 1.26 (0.58 - 1.52) | 112 (89 - 141) | 3.31 (2.65 - 4.13) | 201 (167 - 243) | 5.34 (4.42 - 6.37) | 65 (48- 92) | 1.26 (0.58 - 1.52) |
| Uzbekistan | 100 (62 - 153) | 0.54 (0.35 - 0.81) | 611 (290 - 964) | 1.83 (0.87 - 2.89) | 271 (98- 465) | 3.99 (3.97 - 4.00) | 340 (261 - 441) | 1.83 (1.45 - 2.30) | 2065 (1080 - 2935) | 6.18 (3.25 - 8.80) | 272 (98- 465) | 3.99 (3.97 - 4.00) |
| Vanuatu | 0 (0 - 0) | 0.37 (0.23 - 0.58) | 1 (0 - 1) | 0.40 (0.25 - 0.61) | 10 (3- 23) | 0.15 (0.06 - 0.19) | 1 (1 - 2) | 1.26 (0.99 - 1.59) | 3 (2 - 5) | 1.34 (1.04 - 1.70) | 11 (3- 23) | 0.15 (0.06 - 0.19) |
| Venezuela | 197 (120 - 296) | 1.15 (0.75 - 1.68) | 639 (402 - 941) | 2.34 (1.46 - 3.47) | 129 (87- 189) | 2.10 (1.70 - 2.34) | 665 (524 - 860) | 3.89 (3.15 - 4.81) | 2160 (1767 - 2598) | 7.91 (6.50 - 9.49) | 129 (87- 189) | 2.10 (1.70 - 2.34) |
| Vietnam | 322 (197 - 497) | 0.53 (0.33 - 0.80) | 639 (410 - 945) | 0.63 (0.41 - 0.92) | 35 (18- 56) | 0.47 (0.22 - 0.57) | 1089 (844 - 1419) | 1.78 (1.41 - 2.23) | 2161 (1727 - 2687) | 2.13 (1.71 - 2.63) | 35 (18- 56) | 0.47 (0.22 - 0.57) |
| Yemen | 51 (31 - 80) | 0.47 (0.30 - 0.73) | 375 (179 - 694) | 1.27 (0.64 - 2.29) | 196 (66- 399) | 3.49 (3.23 - 3.78) | 173 (131 - 232) | 1.60 (1.24 - 2.02) | 1265 (710 - 2137) | 4.27 (2.43 - 7.21) | 196 (66- 399) | 3.49 (3.23 - 3.78) |
| Zambia | 31 (19 - 49) | 0.45 (0.29 - 0.68) | 483 (278 - 734) | 2.69 (1.56 - 4.03) | 525 (301- 798) | 5.29 (4.22 - 5.78) | 106 (80 - 140) | 1.52 (1.19 - 1.90) | 1633 (1177 - 2042) | 9.07 (6.52 - 11.26) | 525 (301- 798) | 5.29 (4.22 - 5.78) |
| Zimbabwe | 48 (29 - 73) | 0.54 (0.34 - 0.83) | 382 (203 - 589) | 2.64 (1.43 - 4.07) | 425 (200- 665) | 4.27 (2.61 - 4.99) | 163 (125 - 219) | 1.83 (1.42 - 2.30) | 1294 (700 - 1664) | 8.94 (4.83 - 11.40) | 426 (200- 665) | 4.27 (2.61 - 4.99) |

The AAPC was calculated using 31 years of data from 1990 to 2021 and the table only includes data of 1990 and 2021 for comparative analysis. UI, uncertainty interval. ASR, age-stadardised rate. AAPC, average annual percent change of ASR. CI, confidence interval.
